# Exploring the Sequence-based Prediction of Folding Initiation Sites in Proteins

**DOI:** 10.1038/s41598-017-08366-3

**Published:** 2017-08-18

**Authors:** Daniele Raimondi, Gabriele Orlando, Rita Pancsa, Taushif Khan, Wim F. Vranken

**Affiliations:** 1 Interuniversity Institute of Bioinformatics in Brussels, ULB/VUB, Triomflaan, BC building, 6th floor, CP 263, 1050 Brussels, Belgium; 2 Machine Learning Group, Université Libre de Bruxelles, Boulevard du Triomphe, CP 212, 1050 Brussels, Belgium; 3 Centre for Structural Biology, VIB, Pleinlaan 2, 1050 Brussels, Belgium; 4 Structural Biology Brussels, Vrije Universiteit Brussel, Pleinlaan 2, 1050 Brussels, Belgium; 5 MRC Laboratory of Molecular Biology, Francis Crick Avenue, Cambridge Biomedical Campus, Cambridge, CB2 0QH United Kingdom

**Keywords:** Computational biophysics, Machine learning, Protein sequence analyses, Protein folding, Solution-state NMR

## Abstract

Protein folding is a complex process that can lead to disease when it fails. Especially poorly understood are the very early stages of protein folding, which are likely defined by intrinsic local interactions between amino acids close to each other in the protein sequence. We here present EFoldMine, a method that predicts, from the primary amino acid sequence of a protein, which amino acids are likely involved in early folding events. The method is based on early folding data from hydrogen deuterium exchange (HDX) data from NMR pulsed labelling experiments, and uses backbone and sidechain dynamics as well as secondary structure propensities as features. The EFoldMine predictions give insights into the folding process, as illustrated by a qualitative comparison with independent experimental observations. Furthermore, on a quantitative proteome scale, the predicted early folding residues tend to become the residues that interact the most in the folded structure, and they are often residues that display evolutionary covariation. The connection of the EFoldMine predictions with both folding pathway data and the folded protein structure suggests that the initial statistical behavior of the protein chain with respect to local structure formation has a lasting effect on its subsequent states.

## Introduction

Proteins perform a multitude of functions in organisms. To fulfill their function, a well-defined three-dimensional organization of the protein atoms is often required, with many proteins folding independently into such stable structures^1^. Others need help from chaperones to fold^2^, while some only fold upon binding their interaction partner(s)^3^ or do not fold at all^4^. In all cases, the protein sequence encodes its behavior and, by extension, the environmental context that is required for the protein to fold, whether that is the right temperature and/or pH^5^, another biomolecule or a post-translational modification^6^. Proteins that misfold, for example prions or in amyloid formation^1, 7^, can lead to disease. Different theories about how proteins fold independently have been suggested over the last decades^1, 8–10^, with the view of initial formation of foldons, which provide the right context for the rest of the protein to fold, recently strongly supported by hydrogen-deuterium exchange (HDX) based mass spectrometry (MS) experiments^9, 11^.

Foldons are essentially structural elements that likely form easily through favorable interactions between amino acids close to each other in the sequence. These interactions determine the initial conformational states in the pathway towards the native fold, and provide the context for other residues in the protein to fold themselves. The importance of local amino acid interactions was already pointed out decades ago based on information from folded protein structures^12, 13^. The structure of a protein is, however, an end product of the folding process, and does not provide direct information about where the first local structural elements started to form. To obtain a more accurate picture of such ‘early folding’ residues in proteins, we recently created the Start2Fold database, which collects data from pulsed labelling and related HDX experiments^14^. We showed that the DynaMine sequence-based protein backbone rigidity predictions^15, 16^ give the best results in discriminating early folding residues from other regions of the protein^17^. In addition, we observed that protein regions with higher backbone rigidity tend to preserve this rigidity in evolution^17^.

Experimental early folding data remain difficult to obtain, however, and are only available for specific proteins. We here present EFoldMine, a protein-sequence predictor of early folding residues trained on a set of 30 proteins for which high-quality experimental HDX data is available in Start2Fold. The ‘early folding’ residues in the training set were identified by NMR pulsed labelling experiments, where protein folding is triggered from its unfolded state. These experiments can identify residues that form stable local structure very quickly, on the low millisecond timescale, under kinetic control without fast conformational exchange. Residues are only detected if their backbone amide proton is protected from exchange with water by hydrogen bond formation. Information on the type of local structure that is formed is not available from these experiments. EFoldMine therefore identifies the residues in proteins that are inclined to form structural elements unaided during the very first stage of the folding process, prior to the formation of specific defined interatomic contacts in the folded protein. We show that EFoldMine can provide mechanistic insights into the folding process, and can indicate regions of intrinsically disordered proteins poised to fold. On a proteome scale, the predictions pinpoint many of the residues that create the most interactions in the final folded protein structure, as well as detecting residues that tend to display evolutionary covariation. These observations suggest that early folding events determined by local interactions shape the folding landscape of proteins, so influencing the fold the protein finally adopts.

## Results

### Method performance

The NMR pulsed labelling HDX data that pinpoints the residues where folding starts are difficult to obtain experimentally. The EFoldMine training set encompasses the available high-quality entries from the public Start2Fold database^14^, in total 30 proteins comprising 3398 residues, of which 482 were classified as early folding. As features, EFoldMine uses sequence-based predictions of backbone and side-chain dynamics, as well as secondary structure propensities. These features are incorporated in an SVM with RBF kernel that was cross-validated on sequences stratified by identity (see Methods). To further guarantee the robustness of our method, we limited the dimensionality of the feature vectors, ending up with 25 dimensions for 3398 vectors. The performance measures of EFoldMine obtained through our stratified 27-fold cross-validation are shown in Table 1, while their changes with incrementing features (Table S1), and their full distribution over the leave-one-out cross-validation (Supplementary Fig. S1) are provided as supplementary data. The sensitivity indicates that EFoldMine is able to detect 75% of early folding residues at the cost of over-predicting 25% of the non-early folding ones as false positives. The precision is quite low (36%), but becomes higher if only the most confident 10% or 5% predictions are considered (respectively 45% and 48% of precision).

**Table 1:** Average of leave-one-out stratified cross-validation (27 sets) performance of EFoldMine based on the 30 proteins available in Start2Fold (see Supplementary section 1 for information on the performance indicators).

Parameter	Performance %
Sensitivity (Sen)	73.1
Specificity (Spe)	75.2
Accuracy (Acc)	73.4
Balanced Accuracy (Bac)	74.1
Precision (Pre)	36.1
Matthews Correlation Coefficient (MCC)	35.4
ROC Area Under the Curve (AUC), cutoff 0.169	80.8
Best PPV, 10%, 5%	45.6, 48.8

### Mechanistic insights into protein folding

Experimental studies that reveal details of the protein folding pathway with mechanistic descriptions are difficult to perform and their number remains limited^14^. Based on available data and computational studies that emulate folding, different theories for protein folding have been formulated, such as hierarchical and parallel ‘foldon’ formation^9, 18^. Mechanistically, the process seems to be governed by a balance between local residue interactions, which remain important in folded proteins^19^, as well as topological complexity^20–22^. This determines the order of formation of, or changes in, secondary structure, which in turn modulate the conformational heterogeneity of the protein. We first investigate how the predictions relate to two extensively investigated protein pairs that have a very similar topology but different folding pathways: (i) myoglobin and leghemoglobin, and (ii) proteins L and G.

### Folding of myoglobin and leghemoglobin

**Figure 1:**
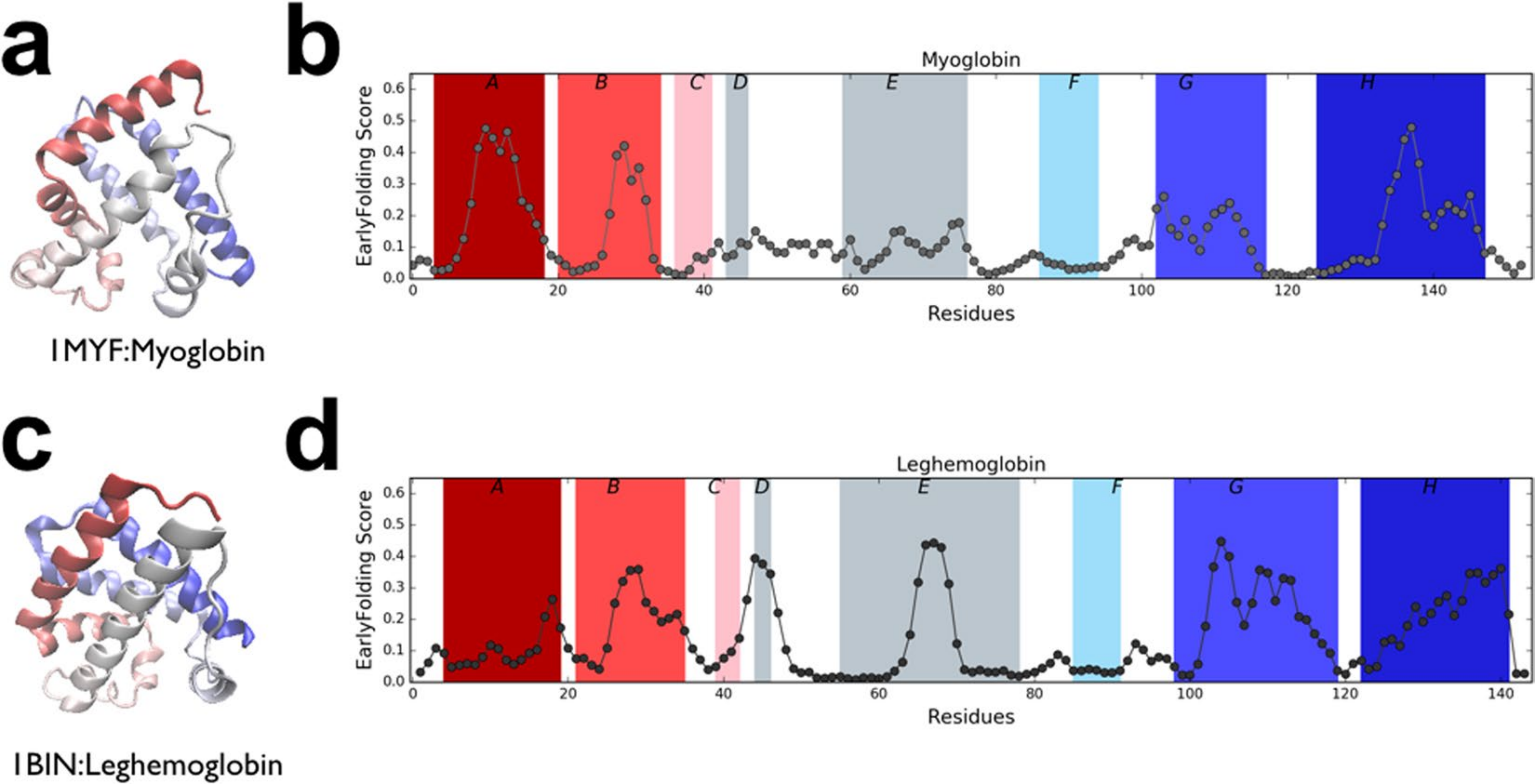
Myoglobin and leghemoglobin in relation to early folding scores. Myoglobin (PDB: 1MYF) (**a**) cartoon representation with helices colored from of N to C terminal and (**b**) full per-residue EFoldMine prediction, with helix regions (A–H) indicated with colors as in a. Leghemoglobin (PDB:1BIN) (**c**) cartoon representation with helices colored from of N to C terminal and (**b**) full per-residue EFoldMine prediction, with helix regions (A–H) indicated with colors as in (**c**).

Myoglobin (PDB:1MYF) is an all-helical protein that is reported to fold through a kinetic intermediate state, with the presence of molten globule-like kinetic intermediate structures^23–25^. These studies also identified that helices A, G and H (Fig. 1) are the first stable secondary structure elements that are formed, with an absence of hydrogen bonds in the helices of the core regions resulting in transient intermediates in the molten globule state. The C-terminal half of helix B is also stabilised early in the folding process^26^. Leghemoglobin (PDB:1BIN) adopts a very similar fold to myoglobin, with a stable helical structure appearing in the G and H helices during folding, but now together with a small region in the center of the E helix, whereas the A and B helices are not stabilized until later^27^. The early folding scores for myoglobin and leghemoglobin were predicted from their primary amino acid sequence, without using heme information, using a jack-knifed version of EFoldMine where the myoglobin sequence was not included in the training set (Fig. 1). In myoglobin, helices A, G, H and the C-terminal half of helix B have high early folding scores compared to the C, D, E and F helices. The predictions therefore agree well with the experimentally validated folding profile of myoglobin, which computational folding studies were unable to reproduce^28^: the high helical content of myoglobin, with its reliance on local contacts, make the study of transition states starting from the final structure very challenging. In leghemoglobin, helices G, H and to a lesser extent B give higher scores and indeed do fold early. Helix A has low scores and does not fold early, in contrast to myoglobin. Interestingly, there is an early folding peak in the centre of helix E, which is also experimentally observed. The overall distribution of the early folding scores of helix E is in line with helices C and F, which have low overall scores (see Fig. 1 and Supplementary Fig. S2). The myoglobin D helix corresponds to a loop region in leghemoglobin, for which no experimental observations could be made^27^. The high early folding scores for this region indicate that it might nevertheless play a role in the folding process. The pattern of significant differences in early folding scores for the helix regions also highlights the dissimilarities between these two proteins in terms of their folding behaviour (Supplementary Fig. S2). The above study relates to sperm whale myoglobin, which raises the question whether the predicted early folding is similar in other organisms. We obtained a multiple sequence alignment for the human, mouse, chicken and zebrafish myglobin sequences and predicted their early folding scores (Supplementary Figs S3 and S4). Despite the low sequence variation between these sequences, there is considerable variation in the early folding scores, with however high values are maintained for helices A, G and H. The exception is a drop in early folding for helix H in zebrafish, which seems to be compensated by increased scores in other regions of the protein (helices C and F/G).

### Folding pathways of proteins G and L

**Figure 2:**
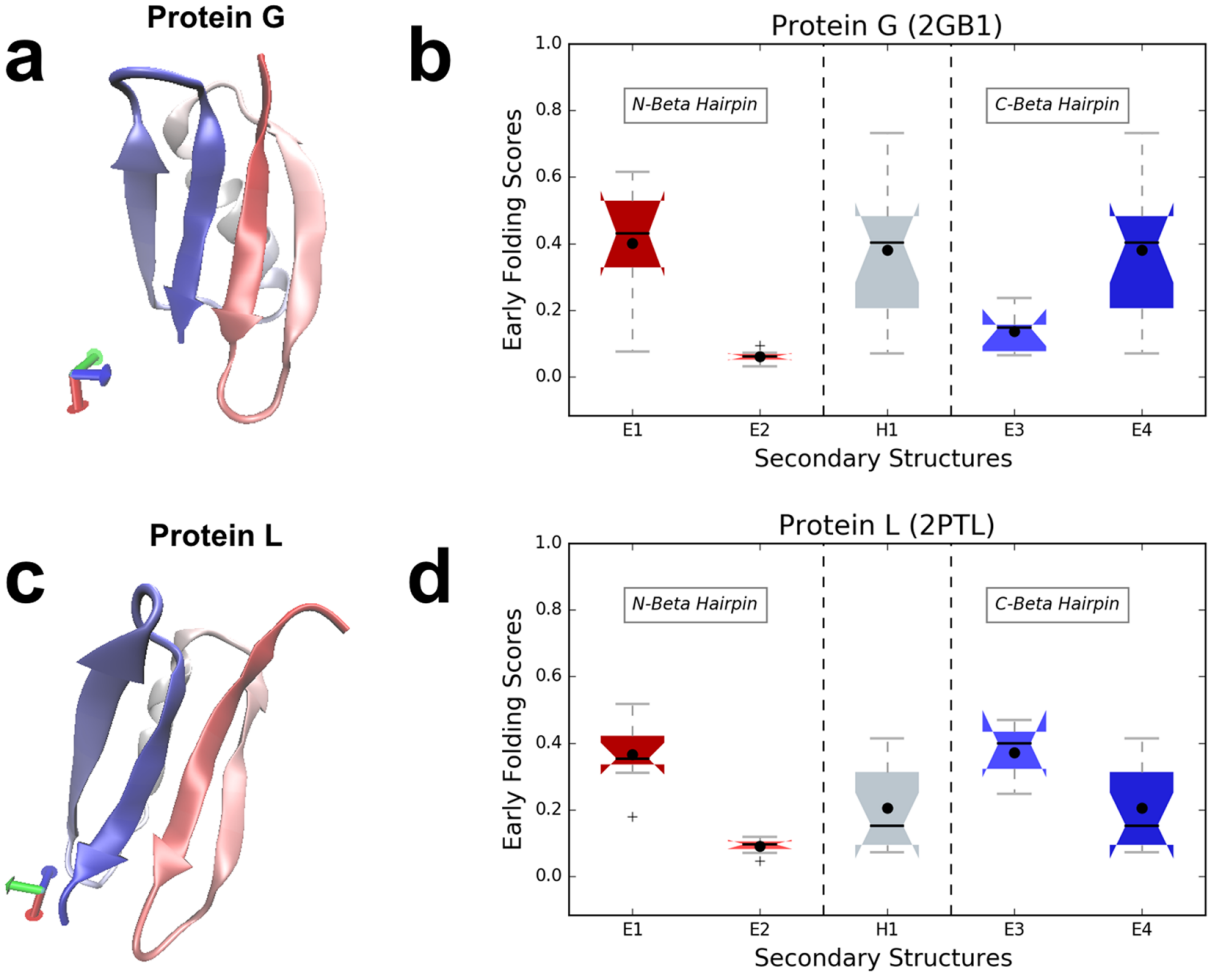
Protein G and L in relation to early folding scores. (**a**) Protein G (PDB:2GB1) with secondary structure elements (E1–E4, H1) indicated, and (**b**) the corresponding distributions of the protein G early folding scores as box plots. (**c**) Protein L (PDB:2PTL) with secondary structure elements (E1-E4, H1) indicated, and (**d**) the corresponding distributions of the protein L early folding scores as box plots.

Protein topology is known to be the governing factor for the overall folding mechanism, but for similar topologies differences in folding can be traced to the residual composition of secondary structures during the folding process. The bacterial surface protein G and protein L have a very similar fold topology, with only minute differences in the orientation of their helices (Fig. 2a,c), despite their low sequence similarity (30%). Extensive experimental and computational studies have shown that their folding mechanisms are different^29, 30^, with an asymmetry in the folding transition state reported in different experimental studies by mutations to alanine in different structural regions^30^. Both experimental and computational results indicate that the C and N-terminal hairpin loops contribute to this difference, with the formation of these secondary structure elements triggering a topologically advantageous folding pathway for protein L, which thus folds into a native-like configuration more quickly than protein G.

The early folding scores for the secondary structure elements of protein G and L (Fig. 2b,d), again calculated with jack-knifed versions of EFoldMine, show that their N-terminal beta hairpins share a very similar pattern, with the first strand having a significantly higher score compared to the second strand (see Supplementary Fig. S5 for significance of the difference between all distributions). On the other hand, the C-terminal hairpin motif composed of strands E3 and E4 is significantly different in protein G and L. The E3 strand in protein G has consistently significantly lower early folding scores compared to protein L (Table S2), while the E4 strand distributions are reversed with a much wider distribution of the values in protein G. The consistently higher early folding scores for the E3 strand in protein L suggest that this strand is primed to form based on local interactions, so enabling the formation of a stable C-terminal hairpin during early folding. Protein G, on the other hand, has higher early folding scores for the E4 strand, but they have a wider distribution, and the E4 strand itself likely experiences more flexibility being located close to the C-terminus. This might complicate the formation of topological connections to create the C-terminal hairpin in protein G, so delaying docking with the helix, which shares similar early folding profiles in both proteins. Overall, these early folding prediction profiles of protein G and L match extensive experimental studies^29, 30^ that suggest that here the C-terminal hairpin is the main topological influence on the folding pathway during the formation of transition state ensembles^31, 32^. Interestingly, mutants designed to increase folding speed and reduce transient structures through manipulation of the E2 strand of protein G^33^ show a correspondingly much higher early folding propensity for this strand (Supplementary Fig. S6).

These examples show that EFoldMine predictions can help in understanding the overall folding mechanism. The residues that are identified are not evident, and are excellent candidates for mutation studies aimed at manipulating or disrupting overall folding.

### Comparison to Mass Spectrometry based studies on MBP and aTS

**Figure 3:**
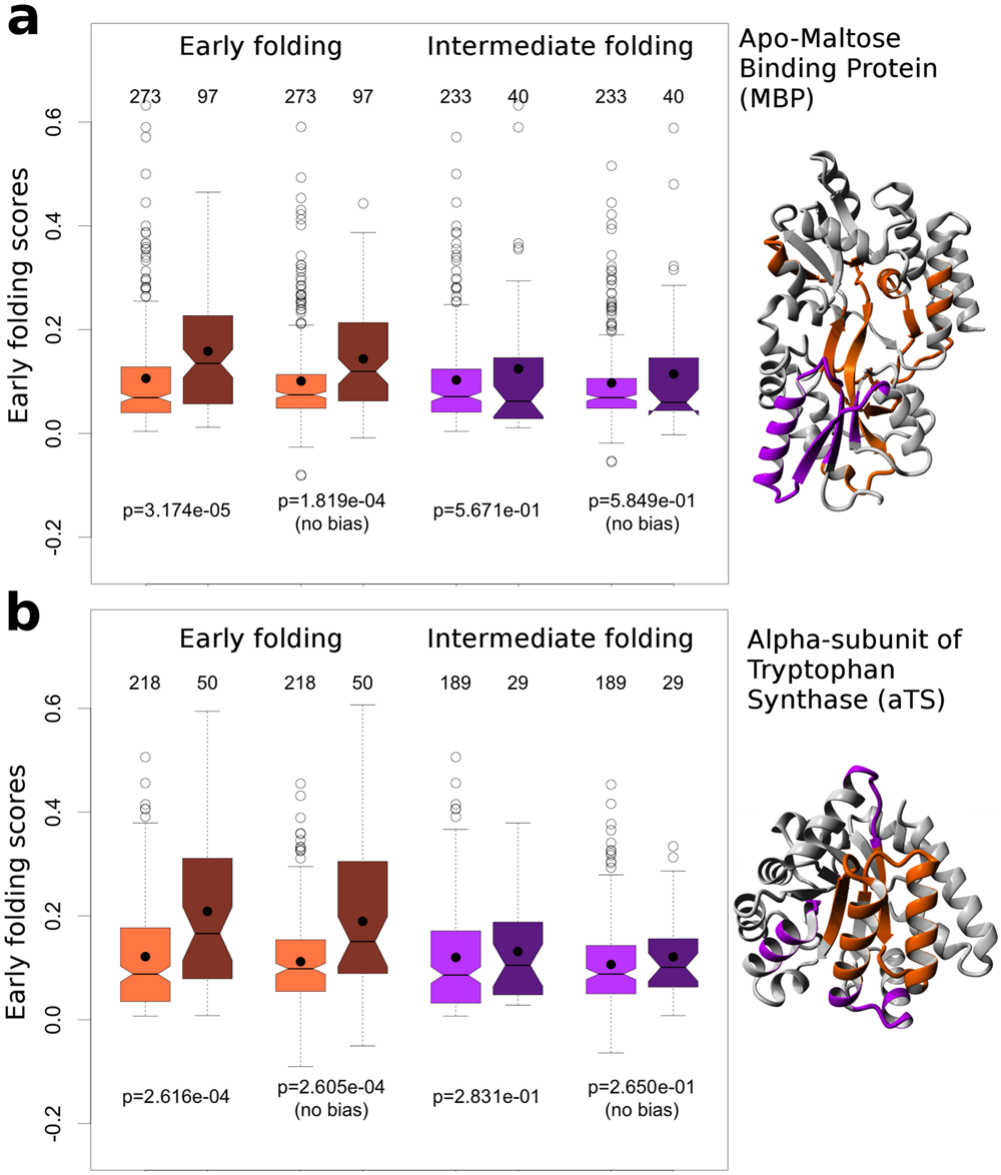
The distribution of the EFoldMine predictions separated by residues experimentally identified by HDX Mass Spectrometry (MS). (**a**) Apo-Maltose binding protein and (**b**) the alpha subunit of tryptophan synthase. The separation by MS data for early (brown) and intermediate (purple) folding residues is shown, with the experimentally identified residues in dark shade, the remaining residues in light shade, and with the amino acid bias-corrected distributions included (no bias). The number of data points is indicated above each distribution, the p-value of the significance of the difference between two distributions below the compared distributions. The protein structures show the early folding (brown) and intermediate folding (purple) regions.

The Start2Fold^14^ database also contains information on early folding regions from HDX-MS experiments for the apo-maltose binding (MBP)^34^ and the alpha subunit of tryptophan synthase (aTS)^35, 36^. These data, which are not part of the EFoldMine training set, are at lower than individual residue-level resolution and also contain information about ‘intermediate’ stages of the folding process, where more complex structures are formed on the pathway toward the native fold. These data therefore provide a valuable independent qualitative comparison point for the EFoldMine predictions. We first subdivided the residues for each protein based on the ‘early’ and ‘intermediate’ classifications from the HDX-MS experiments. The distributions of the early folding predictions for the residues in each class were then compared to the remaining residues (Fig. 3). For MBP, the early folding predictions for the ‘early’ HDX-MS residues are significantly higher than for other residues, with no significance difference for the ‘intermediate’ HDX-MS residues. Both conclusions remain valid when the amino acid bias is removed from the comparison, which is necessary as especially hydrophobic amino acids are more prevalent in early folding regions^17^ (see Methods). In the original MBP HDX-MS paper^34^, the protection from solvent of the ‘early’ set of residues within 0.5 s of starting folding, is attributed to a hydrophobic collapse, whereas the ‘intermediate’ set, emerging at longer timescales (7 s) is due to the first specific structural elements being formed. We therefore further investigated how the relative solvent accessibility (RSA) as determined by DSSP^37^, and the per-residue contact S^2^ parameter^38^ of the final fold of MBP relate to (i) the EFoldMine predictions, (ii) the residues identified by HDX-MS, and (iii) the hydrophobicity as determined by 22 scales. The EFoldMine and ‘early’ HDX-MS residues are both significantly enriched in residues buried in the final fold (low RSA), but this effect disappears when accounting for the amino acid bias (Supplementary Fig. S7). Only the EFoldMine predicted residues are significantly enriched in residues that later form the most extensive contacts (high contact S^2^), even after removing amino acid bias. However, both the EFoldMine and ‘early’ HDX-MS residues give higher hydrophobicity scores in all 22 scales, likely due to the amino acid bias for early folding residues^17^, while the ‘intermediate’ HDX-MS residues are consistently more hydrophilic (Supplementary Table S3). This raises the question whether hydrophobicity scores can actively reproduce the separation between residues with high and low RSA and contact S^2^ values in MBP. We determined the optimal hydrophobicity cutoffs to do this, and all 22 hydrophobicity scales do indeed achieve a significant separation for the RSA values (Supplementary Table S4), also after removing the amino acid bias. This is however not the case for the contact S^2^, where only one scale achieves a significant difference after removing the amino acid bias (Supplementary Table S5). Importantly, these hydrophobicity-based results are not stable overall: for both RSA and contact S^2^ there is considerable variation in the number of points in the high and low distributions, in the difference between their median values, and in the ‘optimal’ hydrophobicity cutoffs between RSA and contact S^2^.

The data for aTS confirm the observations for MBP (Fig. 3). In addition, the relation between the EFoldMine values and high contact S^2^ is even stronger (Supplementary Fig. S8), while the hydrophobicity effect is less pronounced (Supplementary Table S6) and the ‘intermediate’ residues as determined by HDX-MS are also more hydrophobic, in contrast to MBP (Supplementary Table S3). Again, the hydrophobicity scales can separate RSA distributions (Supplementary Table S7), but not the contact S^2^ distributions (Supplementary Table S8), with as in MBP great variability in content of the distributions separated by different hydrophobicity scales.

The EFoldMine predictions, in summary, relate very well to available HDX-MS data for the early folding stage. They also consistently encompass many of the residues that later form the most extensive contacts in the folded protein. The experimental HDX-MS data is not able to do so: these experiments resolve protein fragments, and so likely encompass many non-early folding residues, whereas the HDX-NMR data used for EFoldMine has individual amino acid resolution. Hydrophobicity scales do not provide a valid alternative to EFoldMine or the experimental HDX-MS data: although there tend to be more hydrophobic residues in early folding regions, hydrophobicity in itself is not sufficient to determine the location of early folding residues. This indicates that ‘hydrophobic collapse’, as identified by hydrophobicity values, is too simple a mechanism to explain the complex interactions that happen at this stage of folding.

### Application to an Intrinsically Disordered Protein

**Figure 4:**
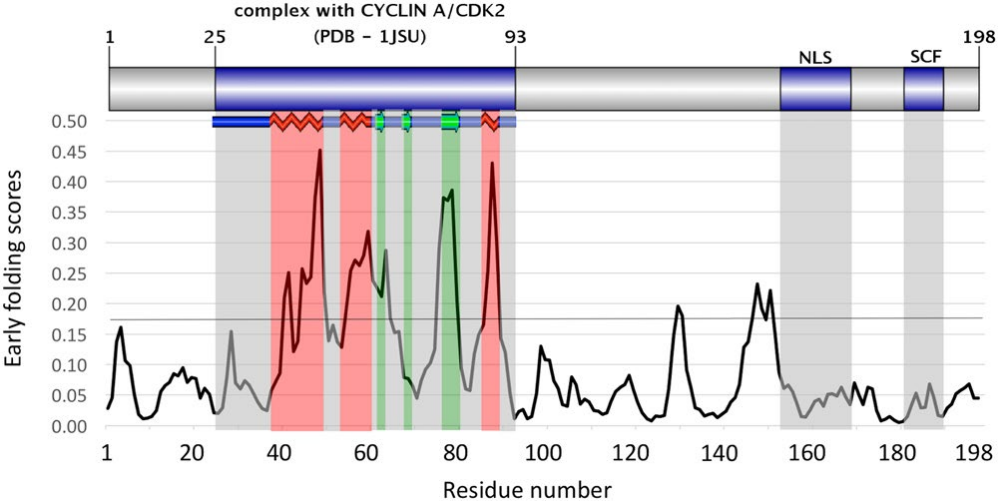
The domain map of p27 ^Kip1^ with the EFoldMine prediction. The blue shaded areas indicate known interaction sites, the red and green shaded areas within the cyclin A/CDK2 interacting region indicate helix and sheet forming segments, respectively.

The cell cycle regulatory human p27 ^Kip1^ protein folds upon binding to its Cdk/cyclin targets. Before binding, p27 ^Kip1^ is at least partially disordered, with parts of its kinase inhibitory domain (KID) exhibiting intrinsic structure that resembles the bound form^39^. The highest EFoldMine-predicted values for this protein correspond exactly to the parts of KID that display strong nascent secondary structure propensity based on NMR studies combined with molecular dynamics simulations^39^, and that are ultimately stabilized in the complex^40^ (Fig. 4). The regions outside the KID, such as the bipartite nuclear localization signal (NLS) motif that usually does not obtain regular secondary structure even when bound by importin-alpha^41^, and a degradation-linked ubiquitination site of the SCF(SKP2) complex that frequently resides within disordered protein segments^42^, have considerably lower prediction values, in line with their intrinsically disordered nature^43^. The correct identification of the pre-structured regions of p27 by EFoldMine suggest that the underlying dynamics features and secondary structure propensities are not only relevant for the folding of globular proteins, but could also advance the prediction of functionally relevant elements within intrinsically disordered proteins (IDPs)^44^, by indicating where the sequence is poised to fold.

### Early folding in relation to the final fold

**Figure 5:**
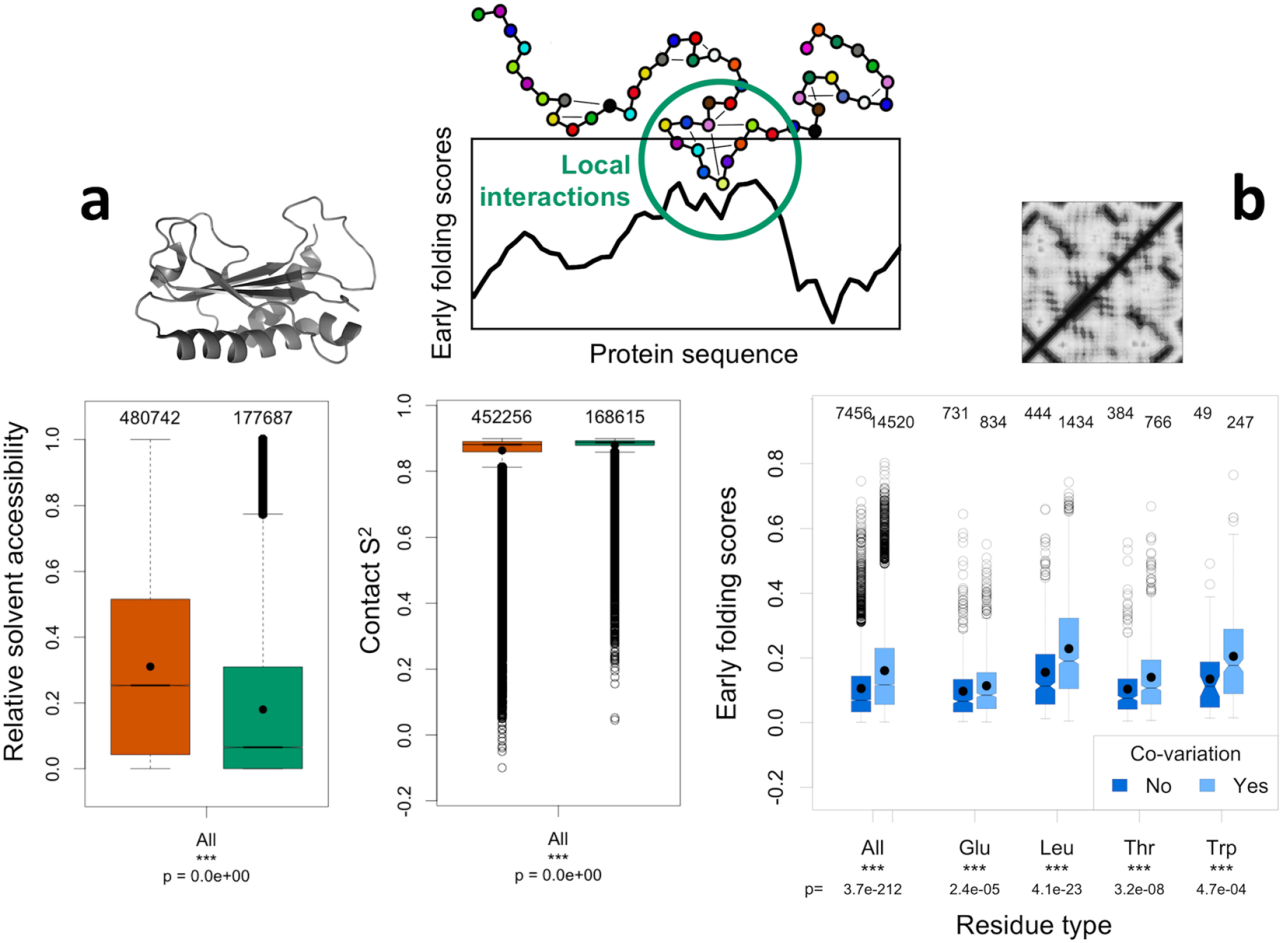
Comparison of early folding scores to structure-related data. The early folding prediction scores (black graph, top) indicate which residues in the sequences will form structure first through local interaction between amino acids (green circle, top). These predictions are compared to (**a**) the relative solvent accessibility and the contact-S^2^ calculated from 2939 non-redundant PDB structures, with significant differences between the distribution of their values for the early folding residues (green) and other residues (brown), and (**b**) residues with evolutionary co-variation signals (light blue), which have higher early folding prediction scores than other residues (dark blue).

The early folding residue predictions are available for any protein with a known sequence, albeit with a lower accuracy than experimental data. From this broader bioinformatics perspective, we can cover a wide range of proteins to address questions concerning the general relation between early folding and the experimentally determined final fold of a protein. The first question we address is whether early folding residues also become key residues in maintaining the final fold. If the early folding residues form local structures that provide the context for the rest of the protein to fold, then they shape the folding landscape: the not-yet folded residues have to interact with these local structures in order to fold themselves. From this assumption, the early folding residues are also essential for the final fold, and should on average become the residues that create the most interactions with other residues in the protein, including long-range ones. Two parameters that reflect such a role in the final fold are the RSA^37^, and the per-residue contact S^2^ parameter^38^, which estimates the rigidity of residues based on the number of heavy atoms in close contact with the backbone amide proton of a residue and the carbonyl atom of the previous residue. Figure 5a shows the distributions of the RSA and contact S^2^ values for the residues in the **Pisces** dataset of 2939 non-homologous (<20% sequence identity) proteins with high resolution (<1.6 Å) x-ray structures, separated on being predicted as early folding (green) or not (brown). For the RSA, there is a highly significant difference between the two distributions, although not all buried residues are necessarily involved in folding: side-chains might be buried later on during the folding pathway, when key structural elements are already formed. A clearer difference with less overlap between the distributions is observed for the contact S^2^ parameter (Fig. 5a), a measure that is in our opinion more indicative of how important a residue is to the fold. The contact S^2^ reflects how many heavy atoms of other residues are close to the backbone of a residue, and it is less likely that such contacts are formed further along the folding process without requiring significant fold rearrangements. The overall differences in distribution from Fig. 5a are also present when the data is subdivided into individual amino acid types: for both the RSA and the contact S^2^ there are significant differences between the distributions of early folding and other residues for all amino acids (Supplementary Fig. S9). Although the EFoldMine predictions will not be able identify all residues that are core to the final fold, they do reliably identify a subset of these residues, and importantly they do so beyond the typical (hydrophobic) structure-forming residues. This supports the concept that early folding residues shape the folding process by providing the initial context for folding through foldons with native-like interactions, which are maintained in the final fold.

The second question is how the early folding predictions relate to the evolutionary co-variation signal derived from multiple sequence alignments and used in the contact prediction field^45, 46^. Such a signal indicates that those residues co-evolved and that they are likely to be spatially close to each other in the (functional) protein fold. Based on the **ContactPred** dataset, we do observe that residues with covariance signals tend to have elevated early folding predictions (Fig. 5b); this tendency is present on the individual amino acid level and is very pronounced for some residues, in particular Cys, Met, Leu and Trp (Supplementary Fig. S10). Early folding residues therefore tend to co-evolve and tend to be part of the core of the final fold, which suggests that in particular the interactions between these residues have to be conserved in order to maintain the protein fold in evolution. This conclusion is in line with our previous observation that regions with high predicted backbone rigidity tend to be preserved in evolution^17^.

### Discussion

EFoldMine is based on carefully curated pulsed labelling HDX data from NMR, and predicts where proteins are likely to form kinetically determined local structural elements very early in the folding process, generally in the low millisecond range. At that time, the conformations proteins adopt are still largely statistically determined, forming highly dynamic ensembles. There is a crucial experimental difference with native exchange HDX experiments, where residues protected from solvent in the native folded state are identified: the hydrogen/deuterium exchange regime as well as existing conformations and their equilibria play key roles in the native exchange setting. We showed earlier that, although there is overlap between the residues identified by ‘pulsed labelling’ and ‘native exchange’ HDX, they form distinct sets^17^. A predictor primed to identify native exchange residues^47^, for example, does not achieve performances of nearly the same level as EFoldMine on the pulsed labelling data (Supplementary Table S9). In times of big data, a concern might be the size of our training data set: first, these early folding data are experimentally difficult to obtain, and we have selected only the data sets of the highest quality to ensure only residues in the very first stage of folding were included. Secondly, we have been careful to avoid overfitting during the training stage while avoiding sequence identity overlap, with the individual predictors trained on each cross-validation set behaving very similarly. In terms of data bias, our training data relates to smaller proteins, but since early folding behavior is local to the protein sequence the effects we predict come from short-range interactions, which should not be different in longer proteins. A shortcoming of the HDX training data is that only the residues that form hydrogen bonds as donor in the early stages of folding are detected, which increases the importance of residues that are part of secondary structure elements and can form stable hydrogen bonds. Residues that might form transient, non-standard local structure are not included in these data. However, the excellent overlap of the predictions with independent HDX-MS data (Fig. 3) shows that they are do pick up where early folding starts, also in larger proteins. While molecular dynamics (MD) is now also capable of simulating the full folding process^48^, the identification of early folding residues from these simulations presents interesting challenges. The simulations have to extend to the longer millisecond timescale, which is a barrier for larger proteins, and the results continue to depend strongly on the force field used^49^. In addition, the definition of what constitutes an early folding residue based on the MD simulation is interesting but not immediately clear. This likely requires an extensive comparison between MD simulations and experimental data from HDX-NMR, with multiple MD simulations from different starting structures required to obtain a statistically sound picture. Unravelling this connection, and the relation to the EFoldMine predictions, would be very interesting and could lead to further insights on the local amino acid interactions that drive early folding.

The heterogeneity of the conformations that a protein might adopt during the folding process makes interpretation of the progressive folding stages very complex. The EFoldMine predictions can provide a piece of this puzzle: comparison with experimental data for the well-studied myoglobin and leghemoglobin (Fig. 1) and proteins G and L (Fig. 2) show salient differences between the early folding propensity of secondary structure elements, which relates well to the experimental observations. Although we predict in essence short-range interactions, not only alpha-helix (Fig. 1) but also sheet formation is picked up (Fig. 2), with some sheets having higher overall early folding propensities than the alpha-helix in case of protein L. In terms of the folding process, it is likely the combination of early folding propensity and the secondary structure type to be formed that matters, and we hope our contribution will contribute a piece to the puzzle by highlighting the regions and/or secondary structure elements that initiate folding. These region are important for folding but that is not necessarily obvious from the final folded state.

The large-scale investigation of the EFoldMine predictions with independent observations for folded proteins shows that early folding residues (i) also tend to be the residues that make the most backbone interactions in the native fold, and (ii) are likely to co-evolve. This hints at their importance for maintaining the protein fold, which has further implications for structural bioinformatics approaches. Such approaches are typically based on structural data for folded proteins at a highly precise atomic level, even though folded proteins represent a restricted subset of the range of behaviors proteins can exhibit. This is strikingly attested by intrinsically disordered proteins^50–52^. The interactions in folded proteins are also highly context-dependent, *i*.*e*. some precise atomic interactions between residues can only be formed because local structural elements are already present. The early folding predictions we present here instead reflect a more statistical view of proteins that is based on local interactions between amino acids. This perspective should greatly assist in structural bioinformatics analyses for more flexible proteins, but also in relation to the folded state, where dynamic and allosteric characteristics are often important for function^53^. We therefore propose that EFoldMine provides information that is complementary and at least partially orthogonal to the precise and defined protein structure data. By providing information about early folding, EFoldMine adds a piece to the protein folding landscape puzzle, and might help to understand how this affects the final folded state. The conformational preferences of early folding regions are further likely relevant in determining whether a downstream path is taken towards native fold or aggregation, and might be very useful in contact prediction as well as computational protein design.

## Methods

All data described in this section are available online via doi:10.6084/m9.figshare.4598047.

### Target

The prediction target is based on a dataset containing 30 sequences from the Start2Fold database^17^, which cover the full range of CATH and SCOP structural protein families (Table S10). For these sequences, with a length varying between 56 and 164 residues and a median length of 121 residues, high-quality experimental pulsed-labelling (or related) HDX data is available at an individual residue level covering a total of 3398 residues. Only the 482 residues classified as ‘EARLY’ in these Start2Fold data were annotated as early folding. These residues are the first to be involved in sufficient local structure formation so that their backbone HN is protected from solvent by a hydrogen bond: these are the residues that EFoldMine predicts. The secondary structure elements of the folded protein where these residues are found indicate, not unexpectedly, a bias towards helix and sheet (comprising about 20–25% of all early folding residues), with other secondary structure elements also covered (Table S11).

### Features

We used 5 kinds of macro-features computed from the protein sequence using various tools. First, we used DynaMine^15, 16^ to predict the backbone dynamics of each protein. After the prediction, we shifted the dynamics values within each sequence constraining their range between 0 and 1, analogously to Pancsa *et al*.^17^. For each target residue at position i we then considered the DynaMine scores falling within the window of flanking residues between i-2 and i + 2. The final DynaMine macro-feature thus consists of a 5-dimensional vector for each target residue. We refer to this score as DYNA in Table S1. We also computed a new set of predictions for side-chain dynamics and secondary structure propensity using a linear regression approach with exactly the same procedure as DynaMine from NMR chemical shift-based estimations of the side-chain dynamics through the side-chain RCI^54^ and the secondary structure propensity from δ2d^55^. This resulted in 3 prediction scores targeting secondary structure formation propensity (alpha-helix, beta-strand and coil) and one score targeting the dynamics of residues side-chain. From each one of these scores we extracted the features by using a 5-residues window (as for DYNA). The final macro-features related to secondary structure propensity and side-chain dynamics are thus each 5 dimensions long and are respectively called HELIX, STRAND, COIL and SIDE in Table S1. The final feature vector used in this study is composed of these 5 macro-features and is 5*5 = 25 dimensions long, resulting in 3398 feature vectors of 25 dimensions, of which 482 are positive hits and 2916 are negative hits. The amino acid type itself is not directly taken into account for the prediction.

### Training

For the training, we used a SVM with RBF kernel (with parameters C = 100 and gamma = 0.04) from the Python scikit-learn^56^ library. The class weight was modified within the SVM optimization to account for the intrinsic imbalance of the positive cases, since early folding residues are expected to account for 5–10% of the total residues^17^. In order to perform a fair validation of our method, the prediction performances were evaluated in strict stratified cross-validation settings. We used BLASTCLUST to stratify the 30 sequences in function of a 25% sequence identity (SI) cutoff at 90% coverage, obtaining 27 disjoint sets to use for cross-validation. We then performed a 27-fold cross-validation by dividing the 3398 vectors in function of the SI sets and averaging the performances scores obtained. Note that for examination of the case studies, we used a predictor version where the respective case study protein was left out of the training set. The estimated probabilities were computed by applying Platt scaling^57^ to the hyperplane distance scores obtained from the SVM. We used these probabilities to compute the ROC curve, from which we inferred the best decision thresholds, which were used to calculate all the binary classification scores: 0.163 for the estimated probabilities.

### Code availability

The EFoldMine code is available from https://www.dropbox.com/s/eslk4bkpflsgiia/code.zip (***note: this is a temporary link***, ***will be replaced when accepted***), for academic use only as a stand-alone package with the following dependencies: Python2.7 including numpy (> = 1.6.1), ScipPy (> = 0.9) and the scikit-learn package (http://scikit-learn.org/stable/install.html).

### Structure dataset

The PDB entries with an X-ray resolution of 1.6 Å or less and with less than 20% shared sequence identity were taken from the PISCES database. The DSSP-determined per-residue relative solvent accessibility (RSA)^37^ and the per-residue contact S^2^ value^38^ were calculated from the PDB structure coordinates using BioPython-based scripts^58^, and the early folding probabilities were calculated from the sequences as reported in the PDB file. This resulted in the **Pisces** dataset with 2939 PDB entries with a total of 3033 chains and 658597 residues.

### Contact prediction

Based on an analysis of the PSICOV dataset^46^, we identified residues that give at least one co-variation signal in the multiple sequence alignment (MSA). We applied EFoldMine to the target sequences in this **ContactPred** dataset (Supplementary Dataset 2) and divided the scores into two classes: ones for which at least one co-variation signal to another residue was found (C) and ones for which no co-variation signal was identified (N). These distributions were then compared on a per-amino acid type and overall basis.

### Distribution comparisons and plot generation

Plot generation was done in R^59^ through custom Python scripts. In the notched box plots, the coloured box shows the interquartile range, the whiskers indicate the maximum value or the respective quartile value times 1.5 the interquartile range, whichever is less. The notch displays a confidence interval based on the median plus/minus 1.57 times the interquartile range divided by the square root of the number of points. If the notches of two boxes do not overlap, this is strong evidence that their medians differ significantly^60^. A filled circle shows the mean of each distribution. The number of data points for each distribution is, for the per-amino acid plots, indicated above the boxes. Distributions were also compared using the Wilcoxon rank-sum test in R^61^, and for the per-amino acid comparisons only p-values that remained significant after applying the Benjamini-Hochberg multiple hypotheses testing correction were retained^62^. Throughout the paper we indicate the retained p-values with *** for a highly significant one less than 0.001, ** a very significant one less than 0.01, and * a significant one less than 0.05.

### Bias correction

The comparisons of distributions over all amino acids are biased because some amino acids are inherently more likely to fold early than others. This bias is strong enough to create significant differences, so we corrected it by subtracting first, for each amino acid type, the median value (relative solvent accessibility, contact S^2^) over all classes for that amino acid type from each actual value, and then renormalizing to the expected value range by adding the median value over all amino acids to their actual value. These bias-corrected distributions are indicated by (no bias) in the plots, and are only to be interpreted relatively, *i*.*e*. to assess the difference between the distributions.

## Electronic supplementary material

Supplementary information
