# Author Correction: Exploring the Sequence-based Prediction of Folding Initiation Sites in Proteins

**DOI:** 10.1038/s41598-019-48219-9

**Published:** 2019-08-15

**Authors:** Daniele Raimondi, Gabriele Orlando, Rita Pancsa, Taushif Khan, Wim F. Vranken

**Affiliations:** 1 Interuniversity Institute of Bioinformatics in Brussels, ULB/VUB, Triomflaan, BC building, 6th floor, CP 263, 1050 Brussels, Belgium; 2 Machine Learning Group, Université Libre de Bruxelles, Boulevard du Triomphe, CP 212, 1050 Brussels, Belgium; 3 Centre for Structural Biology, VIB, Pleinlaan 2, 1050 Brussels, Belgium; 4 Structural Biology Brussels, Vrije Universiteit Brussel, Pleinlaan 2, 1050 Brussels, Belgium; 5 MRC Laboratory of Molecular Biology, Francis Crick Avenue, Cambridge Biomedical Campus, Cambridge, CB2 0QH United Kingdom

Correction to: *Scientific Reports* 10.1038/s41598-017-08366-3, published online 18 August 2017

This Article contains errors in the Methods section under subheading ‘Code availability’.

“The EFoldMine code is available from https://www.dropbox.com/s/eslk4bkpflsgiia/code.zip (note: this is a temporary link, will be replaced when accepted), for academic use only as a stand-alone package with the following dependencies: Python2.7 including numpy (> = 1.6.1), ScipPy (> = 0.9) and the scikit-learn package (http://scikit-learn.org/stable/install.html).”

should read:

“The EFoldMine code is available from https://figshare.com/articles/EFoldMine_code/5649373, for academic use only as a stand-alone package with the following dependencies: Python2.7 including numpy (> = 1.6.1), ScipPy (> = 0.9) and the scikit-learn package (http://scikit-learn.org/stable/install.html).”

