# Supplementary material for: Exploring the Sequence-based Prediction of Folding Initiation Sites in Proteins
Source: Sci Rep. 2017 Aug 18;7:8826. doi: 10.1038/s41598-017-08366-3 (PMC5562875; doi:10.1038/s41598-017-08366-3)
Supplement: Supplementary file 1 — Supplementary information [file 41598_2017_8366_MOESM1_ESM.pdf]

SUPPLEMENTARY DATA

Exploring the Sequence-based Prediction of Folding Initiation Sites in Proteins

Daniele Raimondi[1,2,3,4,†], Gabriele Orlando[1,2,3,4,†], Rita Pancsa[5], Taushif Khan[1,3,4] and Wim F. Vranken[1,3,4,*]

[1] Interuniversity Institute of Bioinformatics in Brussels, ULB/VUB, Triomflaan, BC building, 6th floor, CP 263, 1050 Brussels, Belgium; [2] Machine Learning Group, Université Libre de Bruxelles, Boulevard du Triomphe, CP 212, 1050 Brussels, Belgium; [3] Centre for Structural Biology, VIB, Pleinlaan 2, 1050 Brussels, Belgium; [4] Structural Biology Brussels, Vrije Universiteit Brussel, Pleinlaan 2, 1050 Brussels, Belgium; [5] MRC Laboratory of Molecular Biology, Francis Crick Avenue, Cambridge Biomedical Campus, Cambridge CB2 0QH, United Kingdom.

*To whom correspondence should be addressed.

[†] These authors contributed equally

Supplementary section S1: EFoldMine predictor development

We tested different Machine Learning (ML) approaches from the scikit-learn library, starting from linear models such as Logistic Regression and Ridge Classifier. These simpler models gave inferior results compared to the SVM approach. We also tried to predict every sequence as a whole by using structured-output Machine Learning methods, but the performances were significantly lower. In terms of features, we tried to use the amino-acid composition of the target window (a 20-dimensional vector encoding the frequencies of the 20 types of residues) and the amino-acid sequence itself (encoded in a 20×window_size feature vector with one-hot encoding) but the performances were not significantly improved while the overhead in terms of total size of the feature vectors was substantial.

The features included are DynaMine backbone dynamics (DYNA), sidechain dynamics (SIDE), and secondary structure propensities (HELIX, STRAND, COIL). Their progressive performance changes when incorporating these features is shown in Table S1.

Table S1: Performance changes with incrementing features

Feature	Sen	Spe	Acc	Bac	Pre	MCC	AUC
DYNA	0.718	0.674	0.674	0.696	0.313	0.284	0.774
+HELIX	0.692	0.723	0.713	0.707	0.330	0.307	0.788
+STRAND	0.722	0.754	0.738	0.738	0.353	0.347	0.805
+COIL	0.718	**0.757**	**0.740**	0.738	**0.355**	**0.348**	0.807
+SIDE	**0.731**	0.747	0.731	**0.739**	0.354	**0.348**	**0.808**

The range of performances over all 27 cross-validation sets is shown in Figure S1.

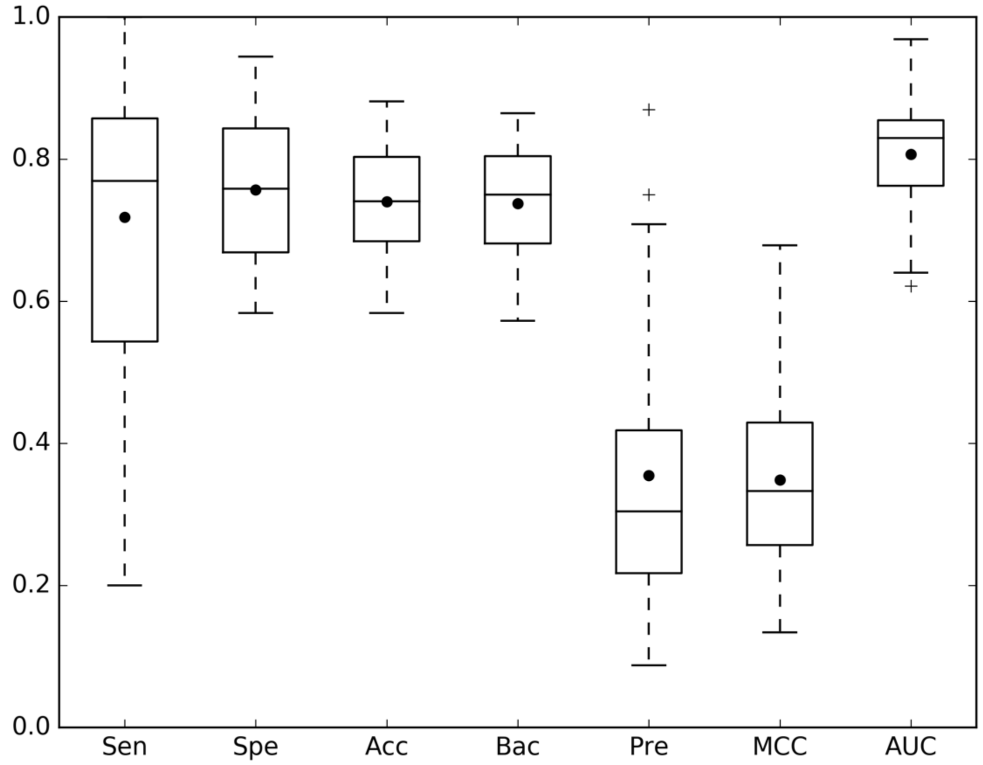

Figure S1. **Cross-validation performances over the early folding dataset.** Sensitivity (Sen), Specificity (Spe), Accuracy (Acc), Balanced accuracy (Bac), Precision (Pre), Matthews Correlation Coefficient (MCC) and Area under the ROC curve (AUC) are indicated.

The performances are calculated by dividing the predictions into correct ones (True Positives and True Negatives, respectively TP and TN) and wrong ones, differentiating between type I and type II errors (False Positives and False Negatives, respectively FP and FN). The scores we use to indicate performances are sensitivity (SEN), specificity (SPE), accuracy (ACC), Balanced Accuracy (BAC), precision (PRE), Area Under the ROC curve (AUC) and Matthews Correlation Coefficient (MCC), which are computed in the following way:

SEN = $\frac{TP}{TP+FN}$ (sensitivity)

SPE = $\frac{TN}{TN+FP}$ (specificity)

ACC = $\frac{TP+TN}{TP+FP+TN+FN}$ (accuracy)

BAC = $\frac{SEN+SPE}{2}$ (balanced accuracy)

PRE = $\frac{TP}{TP+FP}$ (precision)

MCC = $\frac{(TP \times TN)-(FP \times FN)}{\sqrt{(TP+FP)\times(TP+FN)\times(TN+FP)\times(TN+FN)}}$ (Matthews correlation coefficient)

In particular, BAC and MCC are not affected by the unbalancement of the dataset with respect to the positive (early folding) and negative (not early folding) classes, whereas the ACC is strongly influenced by unbalanced data and therefore not a good indicator for the early folding prediction. The AUC relates to the probability that a ML method will rank a randomly chosen positive instance higher than a randomly chosen negative one and it is computed from the Receiver Operating Characteristic curve, which is a plot indicating the performances of a binary classification when the discrimination threshold is varied.

The best PPV at 10% and 5% is the precision compute on the highest ranking 10% and 5% scores obtained by the predictor, assuming that all of them are predicted as positives.

Supplementary section S2: Case studies

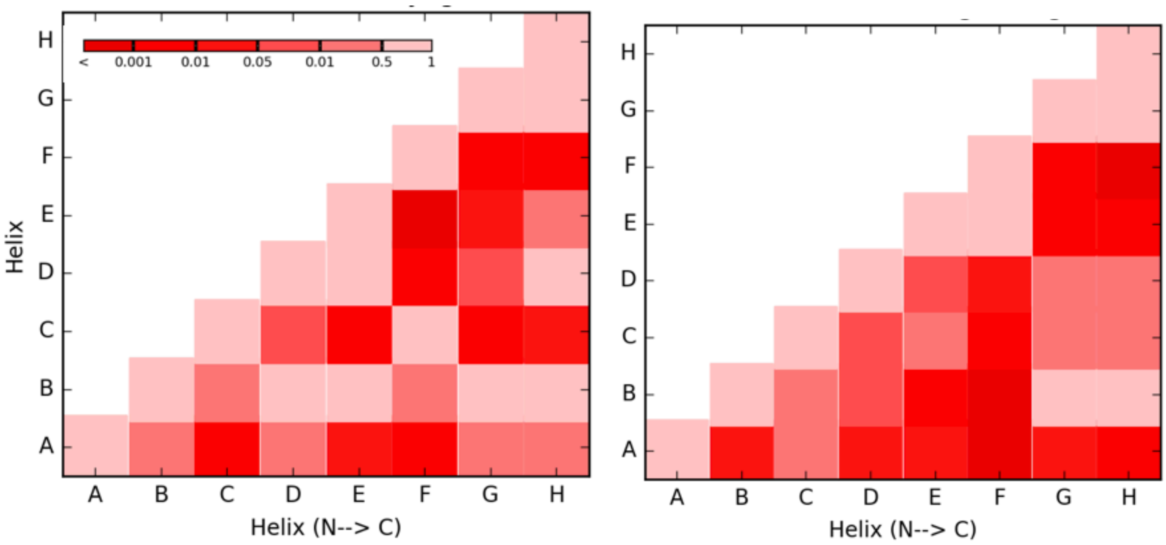

Figure S2. Early folding probability score of each secondary structure (A to H) pair in case of myoglobin (left, PDB: 1MYF) and Leghemoglobin (right, PDB: 1BIN). The difference in the distributions of the early folding scores between each secondary structure was analysed using the Wilcoxon ranksum test. The corresponding P-values are colour coded as shown in the colour bar on the top of figure from low p-value (dark red) to high p-value (light red).

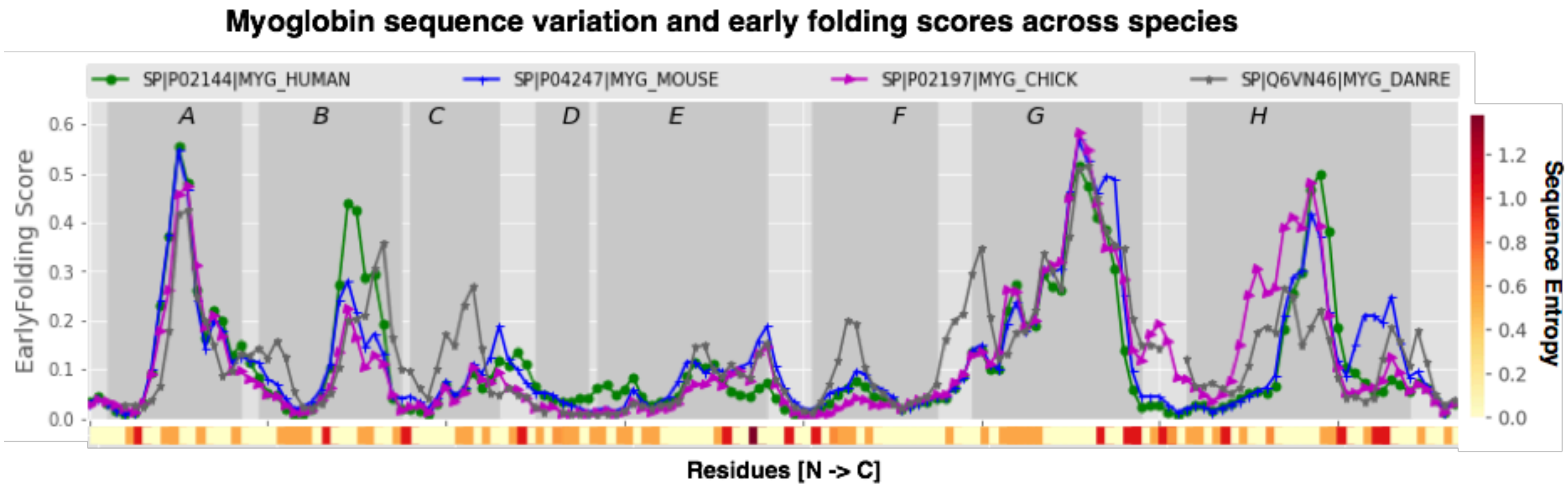

Figure S3. **Early folding score for myoglobin from 4 species.** Human (MYG_HUMAN), mouse (MYG_MOUSE), chicken (MYG_CHICK) and zebrafish (MYG_DANRE) are displayed. The X-axis represents residues from N to C terminal with sequence variation entropy scores colour coded in the bottom. The Y -axis shows the residue wise early folding score for myoglobin from each species. Secondary structure boundaries are shown in grey patches named A to H.

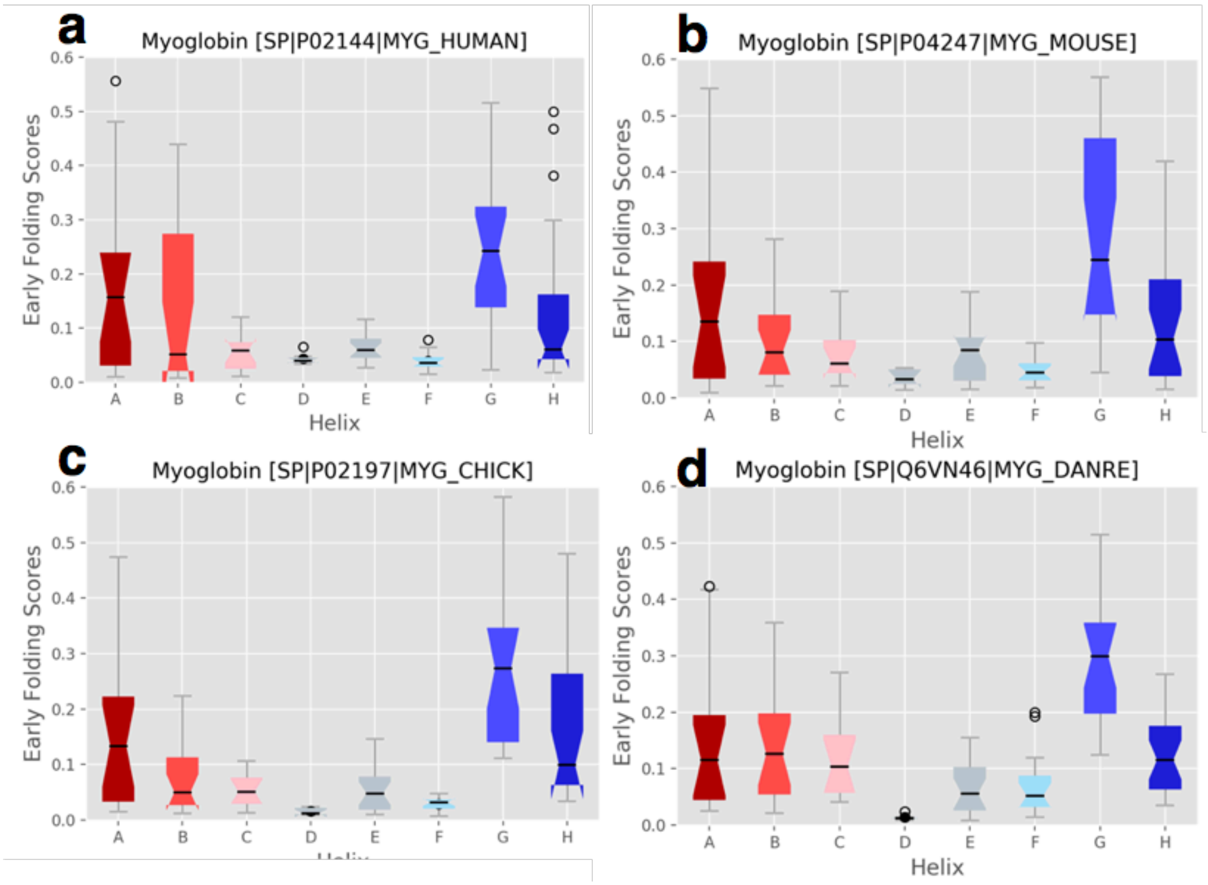

Figure S4. **Early folding score distribution per secondary structure element for myoglobin from 4 species.** Human (MYG_HUMAN), mouse (MYG_MOUSE), chicken (MYG_CHICK) and zebrafish (MYG_DANRE) are displayed. The X-axis represents the helices in myoglobin. The Y -axis shows box plots for the distribution of the early folding scores for each helix in myoglobin.

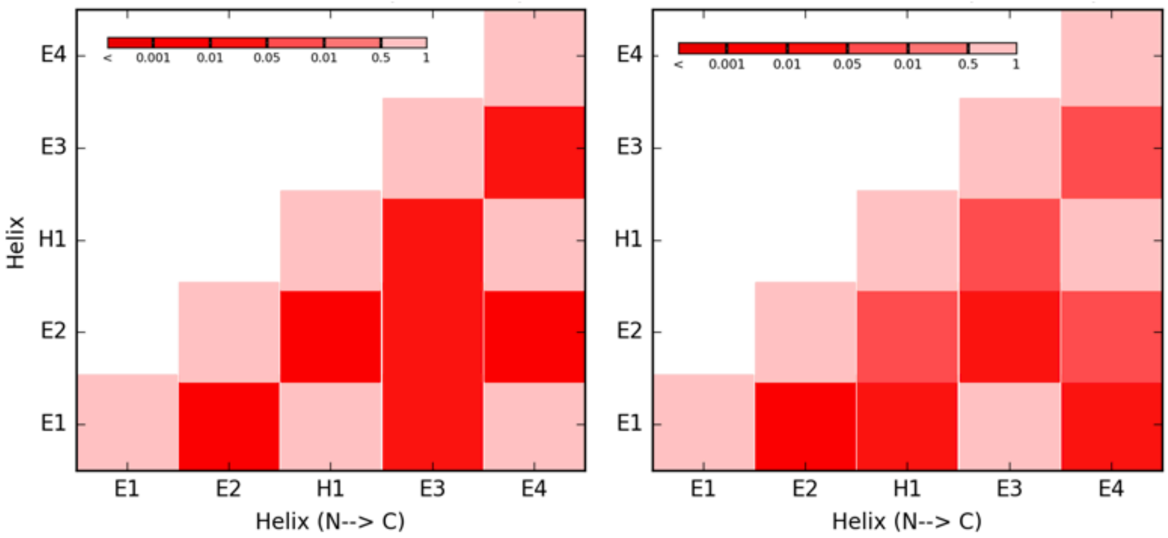

Figure S5. Early folding probability score of each secondary structure (E1 to E4, and H1) pair in case of protein G (left, PDB: 2GB1) and protein L (right, PDB: 2PTL). The difference in the distributions of the early folding scores between each secondary structure was analysed using the Wilcoxon ranksum test. The corresponding P-values are colour coded as shown in the colour bar on the top of figure from low p-value (dark red) to high p-value (light red).

Table S2: Wilcoxon ranksum test p-values for comparing the distributions of early folding prediction scores per secondary structure element in proteins G and L

Secondary structure element	p value
E1	0.5186
E2	0.0633
H1	0.0296
E3	0.0253
E4	0.0296

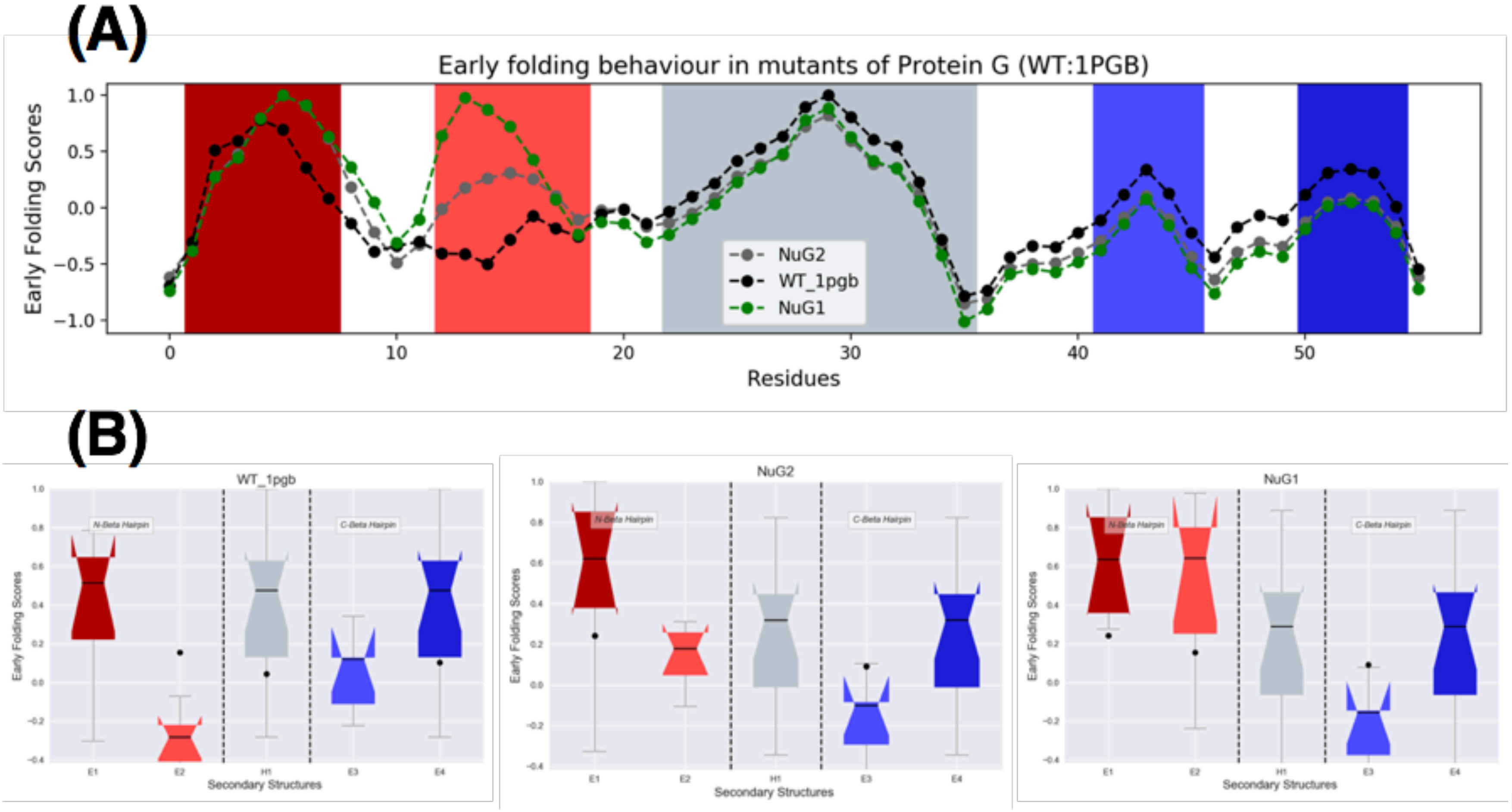

Figure

Figure S6. **Early folding behavior for mutants of protein G.** The wild type protein G (WT_1pgb (“black”)) and its mutants (NuG1 (“green”), NuG2 (“gray”)) are compared residue wise (A) and secondary structure wise (B) from N to C terminal. The mutants are designed to increase folding speed by reducing transient structures, which corresponds, from the early folding perspective, in a much higher early folding propensity for E2.

Supplementary section S3: Comparison to HDX-MS data

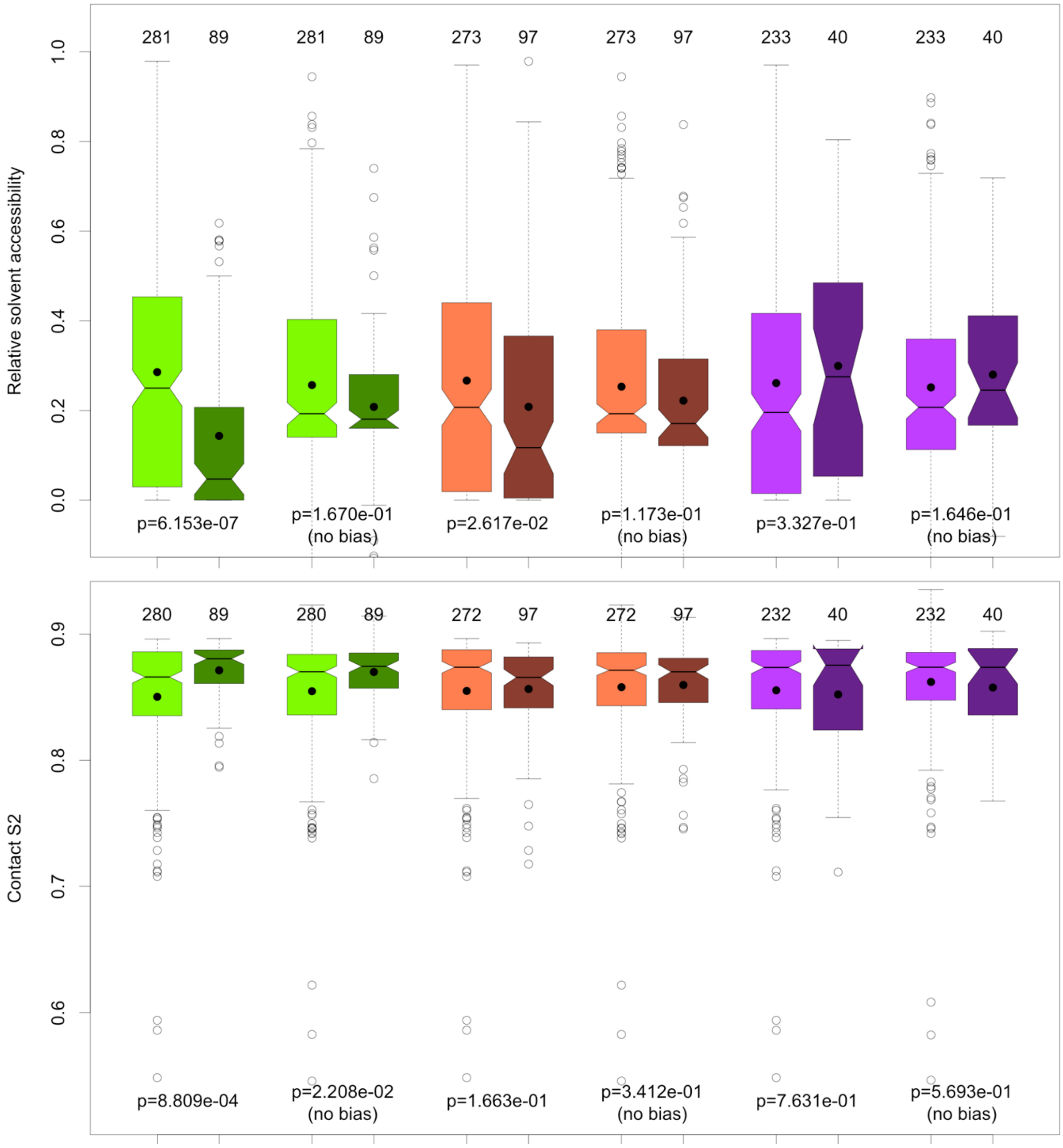

Figure S7: **MBP HDX-MS comparison.** The RSA (top) and contact S^2 (bottom) distributions for EFoldMine predicted early folding residues (green), and the HDX-MS determined early (brown) and intermediate (purple) folding residues for MBP. The number of points per distribution are given at the top, the significance of the difference in distributions at the bottom.

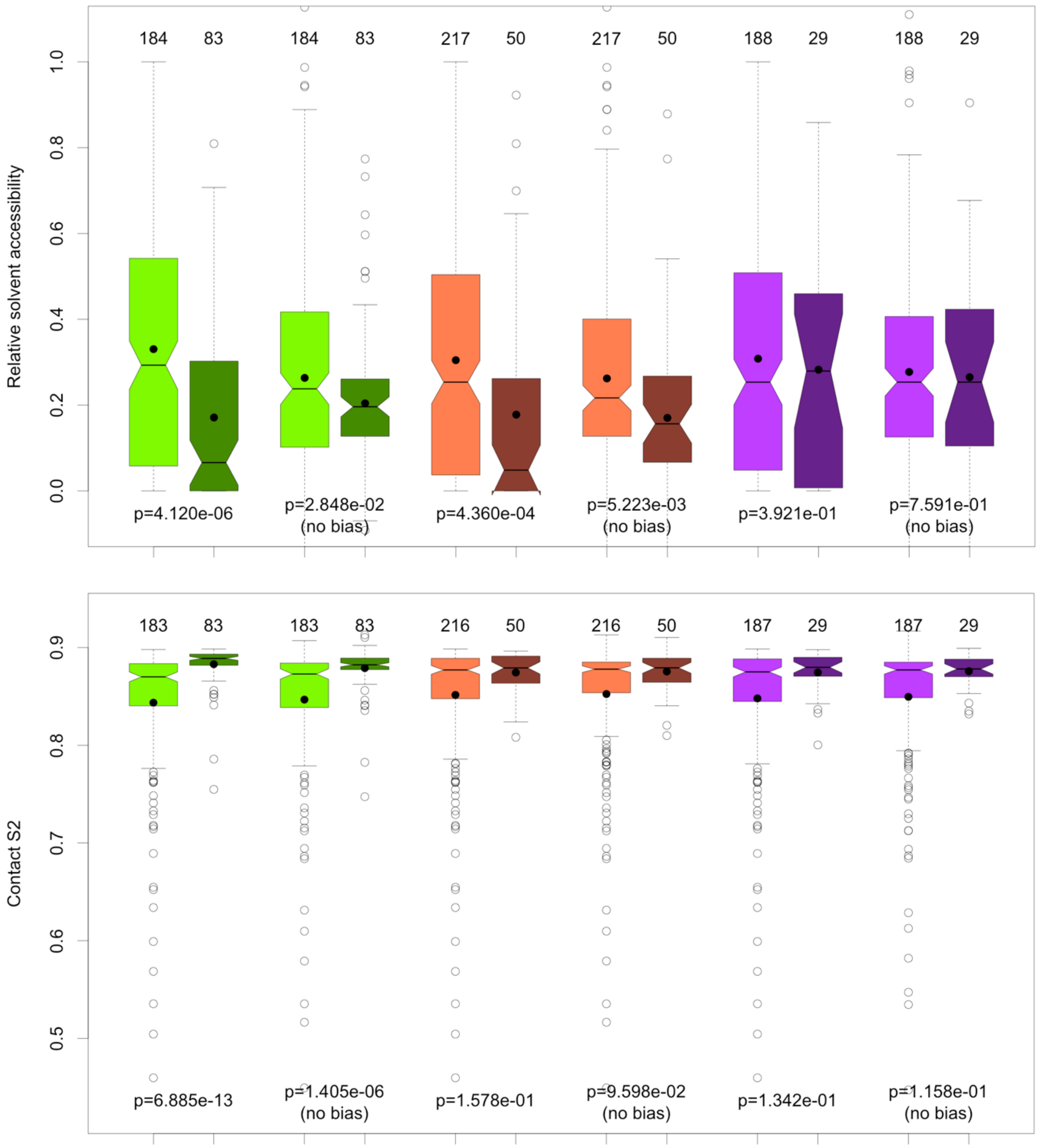

Figure S8: **aTS HDX-MS comparison.** The RSA (top) and contact S^2 (bottom) distributions for EFoldMine predicted early folding residues (green), and the HDX-MS determined early (brown) and intermediate (purple) folding residues for aTS. The number of points per distribution are given at the top, the significance of the difference in distributions at the bottom.

Table S3: The significance of the difference in the distribution of hydrophobicity values (for 22 scales) between residues identified as early folding by EFoldMine, by the 'early' set of HDX-MS and by the 'intermediate' set of HDX-MS for MBP, with values in bold remaining significant after applying a Benjamin-Hochberg correction. The Intermediate-MS set always has a less hydrophobic distribution, the other sets always have a more hydrophobic distribution (compared to all other residues in each case).

Hydrophobicity scale	EFoldMine	Early-MS	Intermediate-MS
hydrophilicity_Hopp	**6.2e-06**	**1.4e-14**	**4.9e-04**
hydrophobicity_Bull$	**7.0e-11**	**5.7e-07**	6.4e-01
hydrophobicity_Parker$	**4.3e-09**	**5.3e-06**	**1.2e-02**
hydrophobicity_Welling$	9.9e-01	**8.7e-04**	5.3e-01
hydropathy_KyteDoolittle	**9.6e-05**	**1.4e-07**	**1.9e-04**
hydrophobicity_Aboderin	**6.2e-09**	**2.5e-10**	**3.8e-03**
hydrophobicity_Abraham	**2.0e-07**	**3.2e-06**	**5.5e-03**
hydrophobicity_Black	**9.1e-07**	**5.0e-16**	**2.2e-03**
hydrophobicity_Chothia	**2.1e-04**	**1.0e-04**	5.6e-01
hydrophobicity_Eisenberg	**1.4e-04**	**4.5e-09**	**6.7e-03**
hydrophobicity_Fauchere	**2.6e-06**	**5.0e-08**	**1.5e-03**
hydrophobicity_Janin	**1.9e-03**	**6.4e-06**	**6.4e-04**
hydrophobicity_Manavalan	**7.6e-08**	**2.0e-06**	8.0e-01
hydrophobicity_Meek	**2.7e-05**	**5.3e-13**	**6.3e-03**
hydrophobicity_Miyazawa	**7.1e-09**	**3.5e-04**	**9.3e-03**
hydrophobicity_Rao	**3.9e-04**	**7.3e-08**	**2.4e-05**
hydrophobicity_Rose	**3.8e-08**	**1.1e-06**	**2.5e-02**
hydrophobicity_Roseman	**3.0e-05**	**2.6e-14**	**1.5e-03**
hydrophobicity_Sweet	**7.9e-13**	**5.6e-15**	4.7e-01
hydrophobicity_Tanford	**1.1e-06**	**1.5e-09**	9.2e-02
hydrophobicity_Wilson	**1.8e-10**	**5.7e-14**	6.7e-01
hydrophobicity_Wolfenden	8.6e-02	**1.9e-08**	1.9e-02

$ Scale reversed, lower scores are for more hydrophobic residues

Table S4: The significance of the difference in the distribution of RSA values (**p**) as achievable by the optimal cutoff for 22 hydrophobicity scales for MBP, with values in bold remaining significant after applying a Benjamin-Hochberg correction. Also indicated are the significance for the amino-acid bias corrected distributions (**p (nb)**), the difference in number of points in the respective 'high' and 'low' RSA distributions (**ΔNP**), the difference in median RSA (**Δmedian**) and the optimal cutoff for the respective hydrophobicity scale (**HS_cutoff**).

**Hydrophobicity scale**	**p**	**p (nb)**	**ΔNP**	**Δmedian**	**HS_cutoff**
hydrophilicity_Hopp	**8.2e-06**	**2.2e-02**	26	0.143	0.002
hydrophobicity_Bull	**1.1e-03**	**1.5e-02**	256	0.136	-0.263
hydrophobicity_Parker	**1.4e-05**	**1.2e-03**	-32	0.137	1.756
hydrophobicity_Welling	**2.3e-03**	**3.5e-02**	42	0.076	-0.154
hydropathy_KyteDoolittle	**1.1e-04**	**3.9e-02**	246	0.154	0.525
hydrophobicity_Aboderin	**5.2e-06**	**2.2e-03**	-134	0.195	4.416
hydrophobicity_Abraham	**1.6e-05**	**8.3e-03**	-144	0.187	0.147
hydrophobicity_Black	**7.2e-06**	**3.2e-03**	-42	0.159	0.518
hydrophobicity_Chothia	**1.5e-04**	**3.4e-02**	106	0.093	0.294
hydrophobicity_Eisenberg	**2.0e-05**	**1.9e-02**	-8	0.135	0.059
hydrophobicity_Fauchere	**2.5e-06**	**1.4e-02**	-62	0.174	0.268
hydrophobicity_Janin	**1.0e-05**	**1.8e-02**	196	0.169	-0.041
hydrophobicity_Manavalan	**4.8e-04**	**8.8e-03**	-102	0.128	12.636
hydrophobicity_Meek	**7.8e-06**	**7.5e-03**	-4	0.146	1.647
hydrophobicity_Miyazawa	**8.9e-07**	**6.3e-03**	94	0.125	5.474
hydrophobicity_Rao	**6.4e-06**	**1.6e-02**	126	0.144	0.919
hydrophobicity_Rose	**1.8e-06**	**6.7e-03**	158	0.147	0.730
hydrophobicity_Roseman	**5.5e-07**	**1.8e-03**	-32	0.197	-0.460
hydrophobicity_Sweet	**5.0e-04**	1.7e-01	-232	0.187	-0.291
hydrophobicity_Tanford	**2.5e-05**	**5.1e-03**	260	0.199	0.413
hydrophobicity_Wilson	**1.7e-05**	**1.2e-02**	-98	0.162	1.173
hydrophobicity_Wolfenden	**9.7e-05**	**1.6e-02**	-76	0.139	-3.945

Table S5: The significance of the difference in the distribution of contact S^2 values (**p**) as achievable by the optimal cutoff for 22 hydrophobicity scales for MBP, with values in bold remaining significant after applying a Benjamin-Hochberg correction. Also indicated are the significance for the amino-acid bias corrected distributions (**p (nb)**), the difference in number of points in the respective 'high' and 'low' contact S^2 distributions (**ΔNP**), the difference in median contact S^2 (**Δmedian**) and the optimal cutoff for the respective hydrophobicity scale (**HS_cutoff**).

Hydrophobicity scale	p	p (nb)	ΔNP	Δmedian	HS_cutoff
hydrophilicity_Hopp	2.4e-02	1.3e-01	-237	0.008	0.633
hydrophobicity_Bull	**1.2e-02**	2.3e-02	-113	0.006	0.039
hydrophobicity_Parker	9.8e-02	1.6e-01	-161	0.007	2.446
hydrophobicity_Welling	**7.4e-03**	3.8e-02	-173	0.009	0.093
hydropathy_KyteDoolittle	**3.2e-03**	3.9e-02	245	-0.013	0.525
hydrophobicity_Aboderin	1.0e-01	1.9e-01	257	-0.008	5.549
hydrophobicity_Abraham	1.8e-01	4.7e-01	-263	0.006	-0.021
hydrophobicity_Black	6.3e-02	2.6e-01	-85	0.006	0.502
hydrophobicity_Chothia	1.6e-01	6.3e-01	-123	0.005	0.264
hydrophobicity_Eisenberg	8.5e-02	7.6e-02	247	-0.007	0.261
hydrophobicity_Fauchere	**1.2e-02**	1.4e-02	-241	0.008	0.105
hydrophobicity_Janin	4.5e-02	3.6e-02	195	-0.009	-0.041
hydrophobicity_Manavalan	6.6e-02	1.9e-01	251	-0.013	13.085
hydrophobicity_Meek	**6.3e-04**	7.9e-03	-169	0.013	0.163
hydrophobicity_Miyazawa	7.3e-02	4.6e-02	93	-0.008	5.474
hydrophobicity_Rao	**4.1e-04**	**2.2e-03**	251	-0.014	0.977
hydrophobicity_Rose	**9.3e-03**	7.7e-02	261	-0.012	0.742
hydrophobicity_Roseman	7.0e-02	3.4e-01	-113	0.006	-0.608
hydrophobicity_Sweet	2.4e-02	1.4e-02	-231	0.007	-0.291
hydrophobicity_Tanford	**1.0e-02**	2.7e-02	259	-0.011	0.413
hydrophobicity_Wilson	1.2e-01	1.2e-01	-209	0.005	0.770
hydrophobicity_Wolfenden	4.9e-02	2.1e-01	-5	0.008	-3.661

Table S6: The significance of the difference in the distribution of hydrophobicity values (for 22 scales) between residues identified as early folding by EFoldMine, by the 'early' set of HDX-MS and by the 'intermediate' set of HDX-MS for aTS, with values in bold remaining significant after applying a Benjamin-Hochberg correction.

Hydrophobicity scale	EFoldMine	Early-MS	Intermediate-MS
hydrophilicity_Hopp	6.0e-01	5.0e-01	6.7e-02
hydrophobicity_Bull[$]	**3.3e-06**	**2.1e-13**	1.4e-01
hydrophobicity_Parker[$]	**6.7e-04**	**2.1e-08**	4.6e-01
hydrophobicity_Welling[$]	**1.2e-04**	**2.6e-09**	2.6e-01
hydropathy_KyteDoolittle	1.6e-01	**8.1e-06**	**7.3e-03**
hydrophobicity_Aboderin	6.9e-02	**2.4e-09**	6.7e-01
hydrophobicity_Abraham	**1.6e-02**	**1.0e-12**	6.6e-02
hydrophobicity_Black	7.9e-02	**1.0e-05**	5.2e-01
hydrophobicity_Chothia	5.4e-02	**1.5e-07**	**3.7e-04**
hydrophobicity_Eisenberg	3.8e-01	**9.8e-07**	3.0e-01
hydrophobicity_Fauchere	**8.8e-04**	**9.6e-12**	8.9e-02
hydrophobicity_Janin	8.0e-01	**5.3e-03**	**2.0e-03**
hydrophobicity_Manavalan	**3.8e-05**	**1.1e-07**	**9.0e-05**
hydrophobicity_Meek	3.4e-02	**1.8e-04**	1.9e-02
hydrophobicity_Miyazawa	**1.8e-05**	**1.7e-10**	**8.4e-05**
hydrophobicity_Rao	3.9e-01	4.9e-02	6.9e-02
hydrophobicity_Rose	**9.6e-04**	**1.5e-06**	**1.3e-06**
hydrophobicity_Roseman	5.8e-01	**5.9e-03**	8.3e-01
hydrophobicity_Sweet	**1.2e-05**	**1.7e-05**	2.6e-02
hydrophobicity_Tanford	2.6e-01	**1.8e-07**	2.3e-02
hydrophobicity_Wilson	**6.3e-04**	**2.3e-02**	**4.1e-05**
hydrophobicity_Wolfenden	4.4e-01	**2.3e-04**	7.8e-01

[$] Scale reversed, lower scores are for more hydrophobic residues

Table S7: The significance of the difference in the distribution of RSA values (**p**) as achievable by the optimal cutoff for 22 hydrophobicity scales for aTS, with values in bold remaining significant after applying a Benjamin-Hochberg correction. Also indicated are the significance for the amino-acid bias corrected distributions (**p (nb)**), the difference in number of points in the respective 'high' and 'low' RSA distributions (**ΔNP**), the difference in median RSA (**Δmedian**) and the optimal cutoff for the respective hydrophobicity scale (**HS_cutoff**).

**Hydrophobicity scale**	**p**	**p (nb)**	**ΔNP**	**Δmedian**	**HS_cutoff**
hydrophilicity_Hopp	**5.1e-04**	1.6e-01	111	-0.200	0.087
hydrophobicity_Bull	**1.2e-03**	**7.7e-03**	-107	-0.207	-0.166
hydrophobicity_Parker	**5.3e-03**	7.1e-02	125	-0.182	1.774
hydrophobicity_Welling	**1.1e-02**	4.2e-02	-79	-0.133	-0.329
hydropathy_KyteDoolittle	**3.8e-05**	5.8e-02	141	0.210	0.558
hydrophobicity_Aboderin	**1.9e-06**	**1.4e-03**	27	0.232	5.071
hydrophobicity_Abraham	**7.6e-04**	**2.4e-02**	75	0.209	0.588
hydrophobicity_Black	**8.4e-05**	4.8e-02	-15	0.220	0.547
hydrophobicity_Chothia	**1.5e-05**	**4.6e-03**	105	0.232	0.327
hydrophobicity_Eisenberg	**1.1e-05**	**1.4e-03**	109	0.234	0.246
hydrophobicity_Fauchere	**2.0e-04**	**2.6e-02**	-19	0.188	0.402
hydrophobicity_Janin	**1.6e-04**	**1.2e-02**	89	0.196	0.019
hydrophobicity_Manavalan	**1.2e-03**	**3.5e-02**	75	0.183	13.044
hydrophobicity_Meek	**7.6e-04**	**4.5e-03**	27	0.193	2.019
hydrophobicity_Miyazawa	**1.2e-04**	**7.9e-04**	-147	0.218	5.351
hydrophobicity_Rao	**1.6e-04**	**2.3e-02**	-61	0.148	0.919
hydrophobicity_Rose	**5.5e-06**	**3.3e-03**	-79	0.217	0.725
hydrophobicity_Roseman	**5.4e-04**	**3.5e-02**	-43	0.193	-0.288
hydrophobicity_Sweet	**1.3e-03**	**6.6e-03**	-81	0.191	-0.105
hydrophobicity_Tanford	**6.2e-05**	**1.4e-02**	135	0.235	0.405
hydrophobicity_Wilson	**1.2e-03**	**1.6e-02**	-3	0.207	1.802
hydrophobicity_Wolfenden	**9.5e-06**	**1.4e-02**	109	0.222	-2.334

Table S8: The significance of the difference in the distribution of contact S^2 values (**p**) as achievable by the optimal cutoff for 22 hydrophobicity scales for aTS, with values in bold remaining significant after applying a Benjamin-Hochberg correction. Also indicated are the significance for the amino-acid bias corrected distributions (**p (nb)**), the difference in number of points in the respective ‘high’ and ‘low’ contact S^2 distributions (**ΔNP**), the difference in median contact S^2 (**Δmedian**) and the optimal cutoff for the respective hydrophobicity scale (**HS_cutoff**).

**Hydrophobicity scale**	**p**	**p (nb)**	**ΔNP**	**Δmedian**	**HS_cutoff**
hydrophilicity_Hopp	**9.8e-03**	1.8e-01	-68	-0.009	-0.153
hydrophobicity_Bull	8.4e-02	5.0e-02	138	-0.006	0.073
hydrophobicity_Parker	**2.3e-02**	8.1e-02	-134	0.010	0.330
hydrophobicity_Welling	1.8e-01	5.7e-01	156	-0.009	0.037
hydropathy_KyteDoolittle	**1.5e-02**	7.9e-03	-74	-0.006	0.491
hydrophobicity_Aboderin	1.3e-01	1.9e-01	152	0.007	5.463
hydrophobicity_Abraham	**2.3e-03**	1.7e-02	-112	0.013	0.303
hydrophobicity_Black	**1.5e-02**	1.5e-01	112	0.008	0.571
hydrophobicity_Chothia	2.6e-01	8.8e-01	62	0.007	0.319
hydrophobicity_Eisenberg	**6.2e-03**	2.1e-02	58	0.011	0.196
hydrophobicity_Fauchere	6.8e-02	1.8e-01	-164	0.011	0.296
hydrophobicity_Janin	**7.5e-03**	1.2e-01	110	0.008	0.034
hydrophobicity_Manavalan	**2.1e-02**	2.2e-02	18	-0.006	12.961
hydrophobicity_Meek	3.7e-02	2.2e-01	-138	0.008	0.744
hydrophobicity_Miyazawa	**3.1e-02**	3.8e-02	-164	-0.011	5.314
hydrophobicity_Rao	1.9e-01	2.8e-01	72	-0.003	0.978
hydrophobicity_Rose	2.3e-01	7.3e-01	96	0.006	0.740
hydrophobicity_Roseman	**5.2e-03**	1.1e-01	88	0.009	-0.094
hydrophobicity_Sweet	**9.8e-03**	2.4e-02	-64	-0.009	-0.086
hydrophobicity_Tanford	**3.8e-03**	4.7e-02	46	0.010	0.304
hydrophobicity_Wilson	**4.8e-03**	3.7e-02	-18	0.008	1.720
hydrophobicity_Wolfenden	**8.7e-03**	1.1e-01	-124	0.011	-3.970

Supplementary section S4: Relation to structure-based parameters

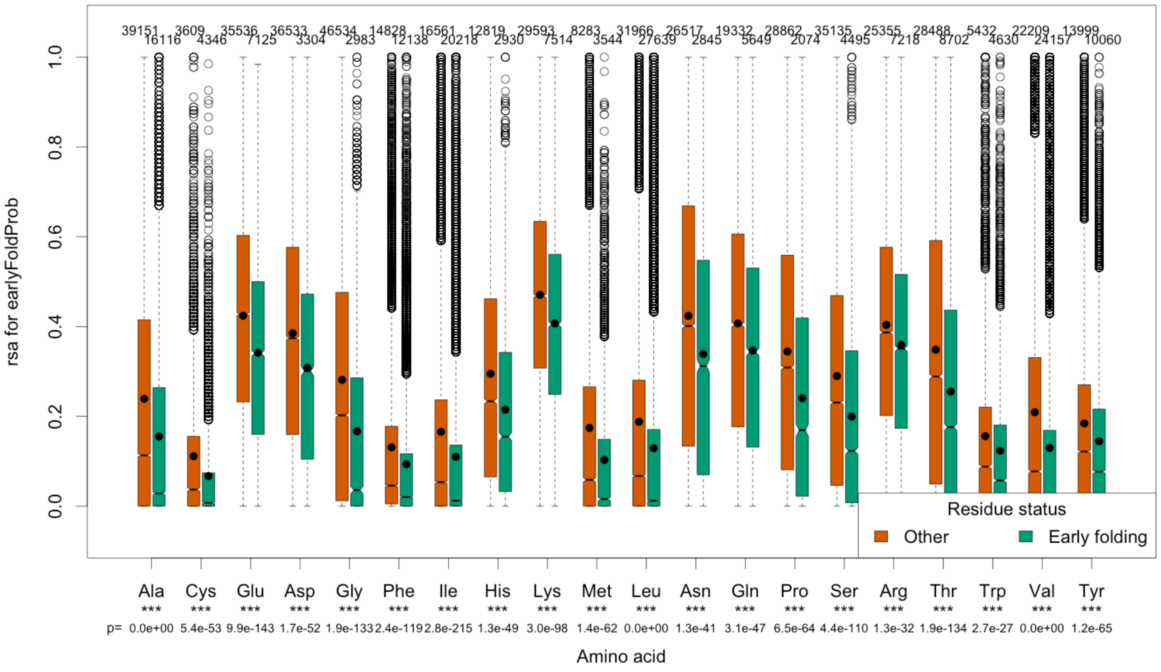

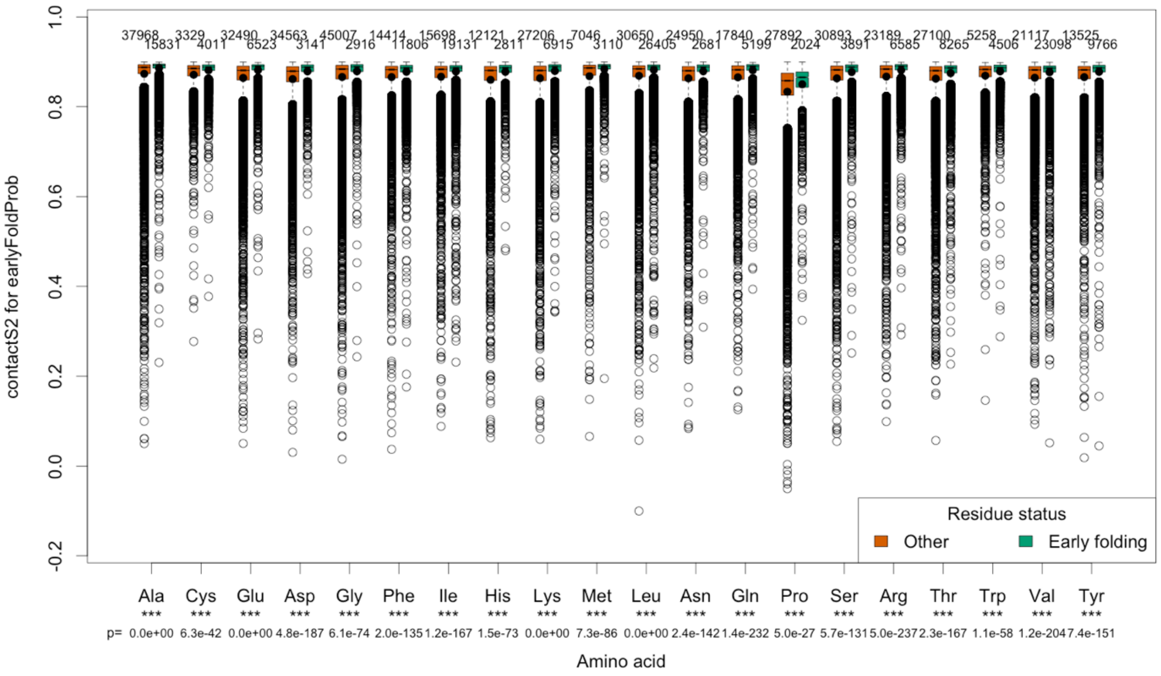

Figure S9. **Folded proteins and early folding.** Per-amino acid distributions for residues in folded proteins from the **Pisces** dataset for relative solvent accessibility (top) and contact S^2 value (bottom) for non-early folding (brown) and early folding (green) residues.

Supplementary section S5: Relation to evolutionary co-variation signal

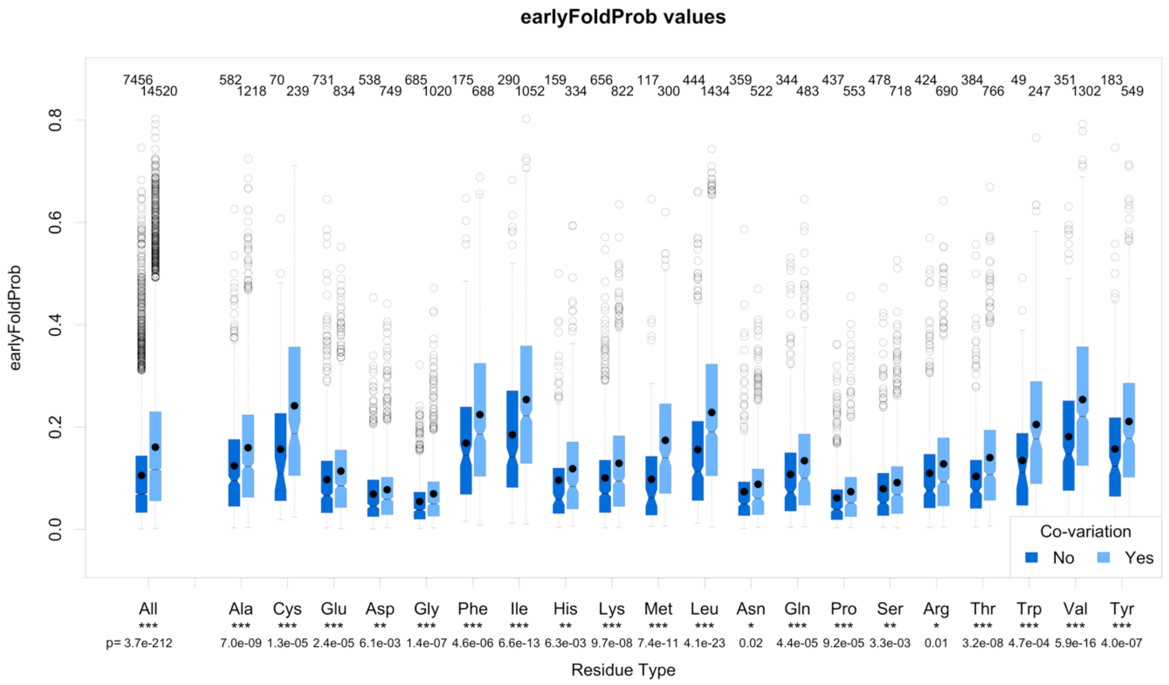

Figure S10. **Co-variation and early folding.** Per-amino acid early folding score distributions for residues that give co-variation signals and ones that do not in the **ContactPred** dataset.

Supplementary section S6: Performance of native-exchange based predictor

Table S9: Performances of a native exchange HDX-based predictor[1] on early folding data.

Sensitivity	0.654
Specificity	0.653
Balanced accuracy	0.653
Precision	0.251
Matthews correlation coefficient	0.225
Area under the ROC curve	0.703

1. Lobanov, M. Y. *et al.* A novel web server predicts amino acid residue protection against hydrogen-deuterium exchange. *Bioinformatics* **29,** 1375–1381 (2013).

Supplementary section S7: Distribution of the folds for representative proteins of the 27 separate training sets.

Table S10: CATH and SCOP protein structure family classifications for the overall fold for representative proteins of the 27 separate training sets.

	Total
CATH	
Mainly Alpha	8
Mainly Beta	8
Alpha Beta	10
Few secondary structures	1
SCOP	
All alpha	5
All beta	6
Alpha and beta (a+b)	9
Alpha and beta (a/b)	3
Small proteins	4

Supplementary section S8: Distribution early folding residues in secondary structure elements as observed in the final fold

Table S11: Distribution of early folding residues for representative proteins of the 27 separate training sets used in the machine learning by secondary structure element in the final fold.

	Total	Early folding	
	Number	Number	Relative percentage
Protein Data Bank (reported)			
Helix (H)	878	178	20.3%
Strand (E)	736	188	25.5%
Coil (C)	1372	78	5.7%
DSSP (calculated)			
Helix (H)	811	174	21.4%
Strand (E)	704	181	25.7%
Coil (C)	643	39	6.1%
H-bonded turn (T)	355	20	5.6%
Bend (S)	328	15	4.5%
3_{10} helix (G)	75	6	8.0%
β-bridge (B)	45	9	20.0%
π helix (I)	10	0	0.0%
Stride (calculated)			
Helix (H)	853	180	21.1%
Strand (E)	751	190	25.3%
Coil (C)	568	30	5.3%
Turn (T)	681	31	4.6%
3_{10} helix (G)	77	8	10.4%
β-bridge (B/b)	40	5	12.5%
π helix (I)	0	0	
